# ${{\mathbb{Z}}}_{2}$ vortex crystal candidate in the triangular *S* = 1/2 quantum antiferromagnet

**DOI:** 10.1038/s41535-026-00894-4

**Published:** 2026-05-26

**Authors:** Jakob Nagl, Kirill Yu. Povarov, Benjamin Duncan, Catharina Näppi, Dmitry Khalyavin, Pascal Manuel, Fabio Orlandi, Jeremy Sourd, Beat Valentin Schwarze, Freya Husstedt, Sergei A. Zvyagin, Oksana Zaharko, Paul Steffens, Arno Hiess, David R. Allan, Sarah A. Barnett, Zewu Yan, Severian Gvasaliya, Andrey Zheludev

**Affiliations:** 1 Laboratory for Solid State Physics, ETH Zürich, Zürich, Switzerland; 2 Dresden High Magnetic Field Laboratory (HLD-EMFL) and Würzburg-Dresden Cluster of Excellence ct.qmat, Helmholtz-Zentrum Dresden-Rossendorf (HZDR), Dresden, Germany; 3 ISIS Facility, Rutherford Appleton Laboratory, Chilton, UK; 4 Institut für Festkörper- und Materialphysik, Technische Universität Dresden, Dresden, Germany; 5 PSI Center for Neutron and Muon Sciences, Villigen PSI, Switzerland; 6 Institut Laue-Langevin, Grenoble, France; 7 European Spallation Source ERIC, Lund, Sweden; 8 Diamond Light Source, Harwell Science and Innovation Campus, Didcot, Oxfordshire, UK

**Keywords:** Materials science, Physics

## Abstract

The prospect of merging the paradigms of geometric frustration on a triangular lattice and bond anisotropies in the strong spin-orbit coupling limit holds tremendous promise in the search for exotic quantum materials. Here we identify a new candidate system to realize such physics, the organic quantum antiferromagnet (CD_3_ND_3_)_2_NaRuCl_6_. We report a combination of thermodynamic, magneto-elastic and neutron scattering experiments on single-crystals to determine the phase diagram in axial magnetic fields **H**∥**c** and propose a minimal model Hamiltonian. (CD_3_ND_3_)_2_NaRuCl_6_ displays an ideal triangular arrangement of Ru^3+^ ions adopting the spin-orbital entangled *j*_eff_ = 1/2 state. It hosts residual magnetic order below *T*_N_ = 0.23 K and a highly unusual *H* − *T* phase diagram including three different incommensurate states. Spin-waves in the high-field polarized regime are described by a Heisenberg triangular lattice Hamiltonian with a potential sub-leading bond dependent anisotropy term *J*_±±_. We argue that the multi-**q** ground state in zero magnetic field is a prime candidate for hosting the ${{\mathbb{Z}}}_{2}$ vortex crystal proposed on the triangular Heisenberg-Kitaev model. (CD_3_ND_3_)_2_NaRuCl_6_ is the first member in an extended family of quantum triangular lattice magnets, providing a new playground to study the interplay of geometric frustration and spin-orbit effects.

## Introduction

Quantum antiferromagnets on a *S* = 1/2 frustrated triangular lattice pose one of the oldest and most influential paradigms in quantum magnetism^1–3^. Even the basic Heisenberg model continues to draw considerable interest, as good material prototypes have only recently become available, finally allowing for concrete comparison to theoretical predictions of exchange renormalization and magnon decay^4–6^. Away from the isotropic limit, the possibility of a quantum spin liquid ground state remains an intensely debated issue^7,8^, with numerous theoretical predictions and only a handful of promising material candidates. Recent experimental work has focused mostly on Co^2+^ ^5,9,10^ and Yb^3+^ ^6,11,12^ systems, with the heavy 4*d* and 5*d* transition metals going largely unexplored despite their prominence in the context of bond dependent interactions in Kitaev-honeycomb systems^13,14^. Thus, combining such bond anisotropies with a frustrated lattice topology poses a promising route to realizing new emergent quantum phases.

One such class of exotic ground states - often stabilized by anisotropy and exchange competition - are the topological spin textures^15^. The triangular Heisenberg antiferromagnet has long been known to host ${{\mathbb{Z}}}_{2}$ vortices as topological excitations^16^, particle-like “twists” in the familiar 120^∘^ three-sublattice order. Recently it was realized that they become thermodynamically stable when Kitaev interactions are invoked^17^. Indeed, even a tiny Kitaev term ∣*K*∣/*J* ≪ 1 is enough to condense them into the ground state, forming an incommensurate (IC) triangular superlattice of ${{\mathbb{Z}}}_{2}$ vortices. This is analogous to the formation of skyrmions in chiral helimagnets^18–21^ or Abrikosov vortices in type-II superconductors^22–24^. These predictions have garnered intense theoretical interest^25–33^, in part because this phase appears to be quite robust in the Heisenberg-Kitaev model and may not require excessive fine-tuning of interactions as in the elusive spin-liquids^8^. An experimental implementation of the ${{\mathbb{Z}}}_{2}$ vortex crystal currently remains open: Only a scant few 4*d*/5*d* compounds have been proposed to realize the parent Hamiltonian^25,26,34^ and none are available in single crystalline form, keeping studies in their infancy.

**Fig. 1:**
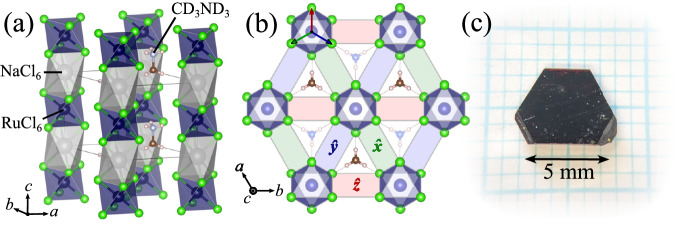
Crystal structure of (CD_3_ND_3_)_2_NaRuCl_6_. **a** Schematic view of the chemical structure, with AA-stacked triangular planes of Ru^3+^ ions. **b** Top-down view of the triangular lattice, emphasizing the three different bond types with parallel-edge RuCl_6_ octahedra. **c** A typical single-crystal sample of (CD_3_ND_3_)_2_NaRuCl_6_.

We address this problem by identifying an entirely new candidate material to host such physics, namely the recently discovered organic quantum antiferromagnetic insulator (CD_3_ND_3_)_2_NaRuCl_6_^35^, crystallizing in a trigonal $P\overline{3}m1$ structure (Fig. 1a). Its magnetism originates from the low-spin 4*d*^5^ Ru^3+^ ions with nearly perfect octahedral coordination, leaving an unquenched *l* = 1 orbital moment that should engender spin-orbital entangled *j*_eff_ = 1/2 degrees of freedom at low temperatures. The material realizes an AA-stacked triangular lattice of RuCl_6_ octahedra in the crystallographic *ab* plane (Fig. 1b), with comparable *in*-plane and *inter*-plane distances *a* = 7.14 Å and *c* = 6.74 Å. As described in the Supplemental Material (Supplementary Figs. S1–4 and Tables T1–3), it undergoes a subtle structural transition to $P\overline{3}$ at *T*^⋆^ ≃ 118 K. However, the ideal triangular coordination of Ru is preserved and Dzyaloshinskii-Moriya terms remain forbidden by symmetry down to the lowest temperatures. The bonds connecting RuCl_6_ octahedra are not of the edge-sharing type as in the famous Kitaev-type honeycomb materials^13^. Instead they display a parallel-edge geometry, nevertheless with two interfering superexchange pathways and three orthogonal bond types (Fig. 1b). A significant Kitaev term has been proposed for such configurations by several authors^14,25,26^, but these claims have yet to be verified experimentally due to a lack of material candidates available in single crystalline form.

In this work, we present a thorough investigation of the magnetic ground states and excitations on single crystals of (CD_3_ND_3_)_2_NaRuCl_6_ through a combination of neutron scattering and bulk thermodynamic probes. We show that the pseudospin wavefunctions are indeed dominated by the ideal *j*_eff_ = 1/2 state. Below *T*_N1_ ≈ 0.23 K, (CD_3_ND_3_)_2_NaRuCl_6_ exhibits a two-step transition into a long-range ordered ground state. The latter turns out to be a complex multi-**q** order with sets of three IC Bragg peaks around the K and H-points, making it a strong contender for the ${{\mathbb{Z}}}_{2}$ vortex crystal phase - with the additional complication of weak AFM inter-plane interactions. In an axial magnetic field **H**∥**c**, we observe a cascade of *seven* field-induced ordered phases with several commensurate-incommensurate transitions. While the commensurate states in the center of the phase diagram may be understood through order-by-disorder selection on a semi-classical Heisenberg model, the three IC phases towards low/high fields point to the relevance of anisotropies. The spin-wave dispersion in the field-polarized regime is captured by a quasi-2D nearest neighbor Heisenberg triangular lattice model with a potential sub-leading bond dependent term *J*_±±_. (CD_3_ND_3_)_2_NaRuCl_6_ is the first member in an entirely new family of quantum triangular lattice magnets based on heavy 4*d*/5*d* transition metals, providing fertile grounds to explore the incommensurate/topological phases emerging from the interplay between geometric frustration and spin-orbit coupling.

## Results

### Basic properties and single-ion physics

**Fig. 2:**
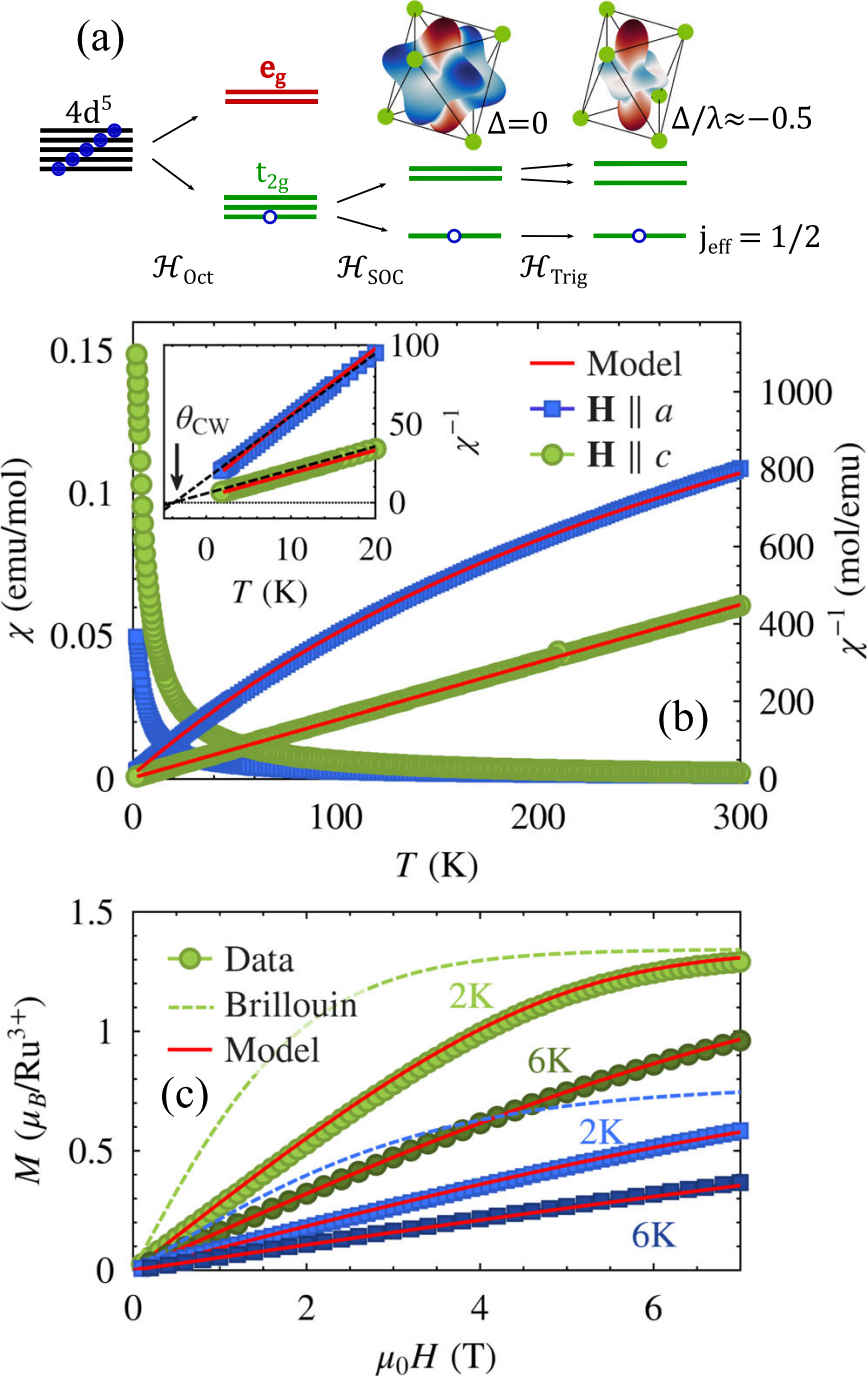
Single-ion physics and mean-field correlations in (CD_3_ND_3_)_2_NaRuCl_6_. **a** Schematic of the energy spectrum for a single Ru^3+^ ion. The free-ion multiplet is split by the cubic crystal electric field, the spin-orbit coupling *λ* and a trigonal distortion Δ. An inset visualizes the spatial shape of the pseudospin wavefunctions, both for the ideal cubic case (Δ = 0) and for the parameters Δ/*λ* ≈ −0.5 relevant to (CD_3_ND_3_)_2_NaRuCl_6_. Red/blue color indicates spin up/down of the hole, mixed together in the spin-orbital entangled *j*_eff_ = 1/2 ground state. **b** Magnetic susceptibility for a small probing field *μ*_0_*H* = 0.1 T along the principal crystallographic axes. The inset shows a low-temperature Curie-Weiss fit. **c** Magnetization curves at various temperatures (markers), along with Brillouin function expected in absence of two-ion correlations (dashed lines). Red lines in (**b**, **c**) represent the calculated magnetic response based on the single-ion + mean-field model discussed in the text.

In Fig. 2b, we present the uniform magnetic susceptibility of (CD_3_ND_3_)_2_NaRuCl_6_ along the principal crystallographic axes. For **H**∥**c** we observe a linear Curie-Weiss behavior up to room temperature in the inverse susceptibility, whereas for in-plane fields, ${\chi }_{ab}^{-1}(T)$ exhibits a strong non-linear bending, indicative of crystal-field effects. The data exhibit a pronounced easy-axis anisotropy, reaching *χ*_*c*_/*χ*_*ab*_ ~ 2.3 in the low-temperature limit. This is likewise reflected in the effective moments ${\mu }_{{\rm{eff}}}=g\sqrt{S(S+1)}{\mu }_{{\rm{B}}}$, pointing to anisotropic *S*_eff_ = 1/2 degrees of freedom with *g*_*c*_/*g*_*ab*_ ~ 1.6, typical for systems with an unquenched orbital contribution. A simple Curie-Weiss analysis in the range 10−40 K results in an average Weiss temperature of *θ*_CW_ ≈ −3.8 K (see Table 1 for details), indicative of moderate antiferromagnetic interactions. Note the limited fitting range (chosen empirically to remain below the temperature at which curvature in ${\chi }_{ab}^{-1}$ becomes apparent), which permits only a rough estimate of *θ*_CW_ and highlights the need for a comprehensive treatment of the single-ion physics (see below). Magnetometry data on the same sample (see Fig. 2c) begin to show significant deviations from a non-interacting Brillouin function below ≲10 K, consistent with an onset of short-range spin correlations.

**Table 1:** Fit results for a Curie–Weiss analysis in the temperature range 10 K ≤ *T* ≤ 40 K

	*θ*_CW_ (K)	*μ*_eff_ (*μ*_B_)	*g*
**H**∥*a*	−3.82	1.42	1.64
**H**∥*c*	−3.93	2.32	2.68

**Fig. 3:**
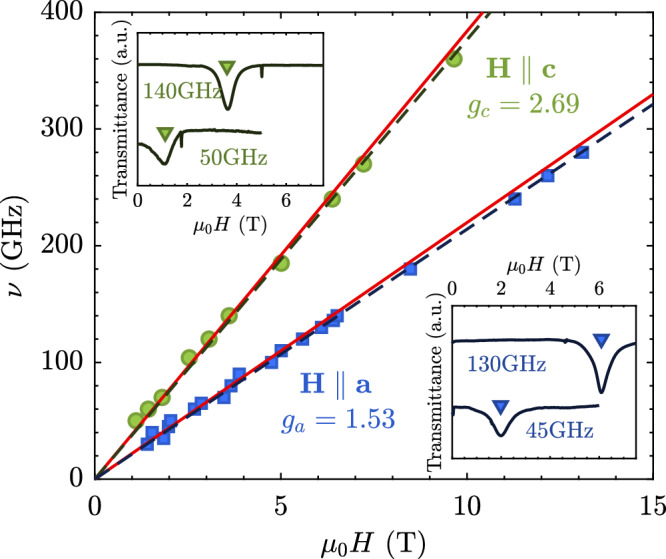
Frequency-field diagram of ESR excitations measured at *T* = 1.5 K for both H∥c (green) and H∥a (blue) field orientations. Dashed lines represent linear fits to the function *hν* = *gμ*_B_*μ*_0_*H*, while solid red lines show calculations based on the single-ion + mean-field model discussed in the text. ESR spectra at selected frequencies are presented as insets. Characteristic dips in transmittance correspond to the Larmor resonant field at given frequency, while sharp features are caused by the *g* = 2.00 DPPH marker.

More precise *g*-factor values are obtained from electron spin resonance (ESR) measurements. For both longitudinal and transverse field geometries, we observe a single resonance line at *T* = 1.5 K, as illustrated in Fig. 3—a rare example of resonance in a strongly correlated system of Ru^3+^ magnetic ions ^36^. The frequency-field dependence is linear, suggesting simple Larmor precession at these temperatures. Fits to *hν* = *gμ*_B_*μ*_0_*H* yield *g*_*ab*_ = 1.53(1) and *g*_*c*_ = 2.69(1), nicely matching our estimates from susceptibility. We detect no apparent temperature dependence of the *g*-factors, as confirmed by ESR data up to *T* ~ 90 K (see Supplementary Fig. S6). Likewise, the spin gap in the field-polarized regime—extracted from heat capacity and inelastic neutron scattering— agrees well with *g*_*c*_ down to dilution temperatures (see below).

For Ru^3+^ transition metal ions with 4*d*^5^ electronic configuration, the octahedral crystal field results in a single hole residing in the *t*_2*g*_ manifold, forming a spin *S* = 1/2 and an effective orbital moment *l*_eff_ = 1. These states are further split by the strong spin-orbit coupling *λ* ~ 0.15 eV^37^ and by a potential trigonal distortion Δ. This is borne out in the following local single-ion Hamiltonian

1 $${\mathcal{H}}=\lambda \hat{{\ell }}\cdot \hat{\mathbf{S}}+\Delta {\hat{{\ell }}}_{z}^{2}-{\mu }_{{\rm{B}}}{\bf{H}}\cdot \hat{\mathbf{M}}$$

where $\hat{\mathbf{M}}=2\hat{\mathbf{S}}-k\hat{{\ell }}$ is the on-site magnetization of the hole and *k* ≲ 1 accounts for a small reduction in orbital moment due to anion hybridization^38^. In the cubic limit ∣Δ/*λ*∣ ≪ 1, the ground state is a spin-orbital entangled *j*_eff_ = 1/2 Kramers doublet with equal weights on all three *t*_2*g*_ orbitals^39^.

The Hamiltonian parameters can be extracted from a global fit to the susceptibility, magnetization and ESR data. In order to account for incipient two-ion correlations, we introduce a Weiss molecular field **H** → **H**_ext_ + **H**_MF_ and solve the resulting eigenvalue problem self-consistently. We obtain *λ* = 153.3 meV, Δ = − 77.9 meV and *J*_MF_ = 1.71 K (for details, we refer to Supplemental Fig. S5 and Table T4), where the latter is a mean-field exchange parameter setting the strength of the molecular field. Our fit results are shown in Fig. 2 as red lines, confirming excellent agreement among different probes. With Δ/*λ* ≃ −0.51, we are still within the spin-orbital entangled regime. The ground state wavefunctions remain dominated by the ideal *j*_eff_ = 1/2 state, though the magnetization density acquires a notable easy-axis character through the trigonal distortion, with slightly more weight on the *ℓ*_*z*_ = ± 1 states (see Fig. 2a). We note such a distortion corresponds to a slight elongation of octahedra along the *c*-axis within a point-charge picture, in agreement with the refined chemical structure. The data are well described by a mean-field exchange constant *J*_MF_ of Heisenberg type—which would correspond to *θ*_CW_ ≈ − 2.6 K—with no signs of XXZ anisotropy within estimated standard deviations. Given the above trigonal distortion, we expect the dominant superexchange scale to be of Heisenberg type^40^, with potential bond anisotropies only taking a sub-leading role. But as we discuss below, even a small bond-dependent term can induce qualitatively new physics on the triangular lattice^17,25^.

### Calorimetry

**Fig. 4:**
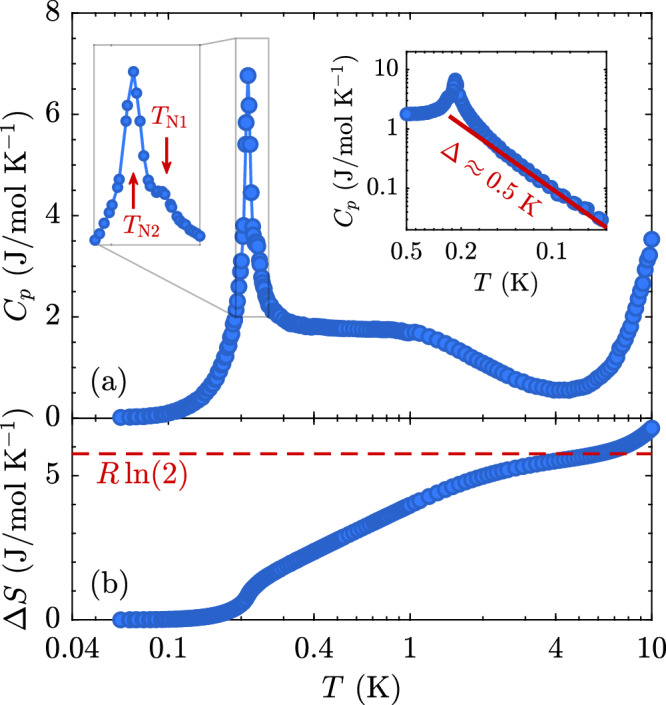
Temperature dependence of specific heat in (CD_3_ND_3_)_2_NaRuCl_6_. **a** Measured heat capacity *C*_*p*_(*T*) in zero magnetic field, exhibiting a two-step transition to magnetic long-range order. Left inset: Zoom-in on the critical region, showing two separate anomalies at *T*_N1_ and *T*_N2_ K. Right inset: Arrhenius plot depicting the activated behavior in the ordered state. **b** Total entropy change Δ*S*(*T*) obtained through numerical integration of the specific heat. Note the plateau at *R*ln(2), reflecting the separation of energy scales between magnetic and lattice degrees of freedom.

In Fig. 4a, we show the temperature dependence of the specific heat capacity in (CD_3_ND_3_)_2_NaRuCl_6_ (note the logarithmic scale). We focus on the low-temperature regime *T* ≲ 4 K, where the lattice is frozen and only magnetic degrees of freedom remain. There is a broad upturn in *C*_*p*_(*T*) around *θ*_CW_, which we associate with the onset of short-range magnetic correlations. It develops into a plateau between 1 K and 0.2 K, followed by a sharp anomaly indicative of a phase transition to magnetic long-range order. The latter takes place in two steps: Zooming in close to the critical region reveals both a shoulder feature at *T*_N1_ = 0.23 K and a sharp peak at *T*_N2_ = 0.215 K. In the ordered phase, the heat capacity fits either to a (gapless) power-law *C*_*p*_ ∝ *T*^*x*^ with *x* ≈ 4.5, or to an activated behavior *C*_*p*_ ∝ *e*^−Δ/*T*^ with Δ ≈ 0.5 K. While such a strong power-law exponent seems rather unphysical, a small excitation gap of order ~0.5 K is consistent with the magnetic energy scale. This points to a gapped ground state, perhaps hinting at some anisotropy terms that break the O(3) symmetry in spin-space. The total change in entropy Δ*S* = ∫*C*_*p*_/*T* (see Fig. 4b) is extracted through numerical integration of the heat capacity data. It reaches a plateau around 4 K, nicely extrapolating to *R*ln(2) as expected for *j*_eff_ = 1/2 degrees of freedom.

**Fig. 5:**
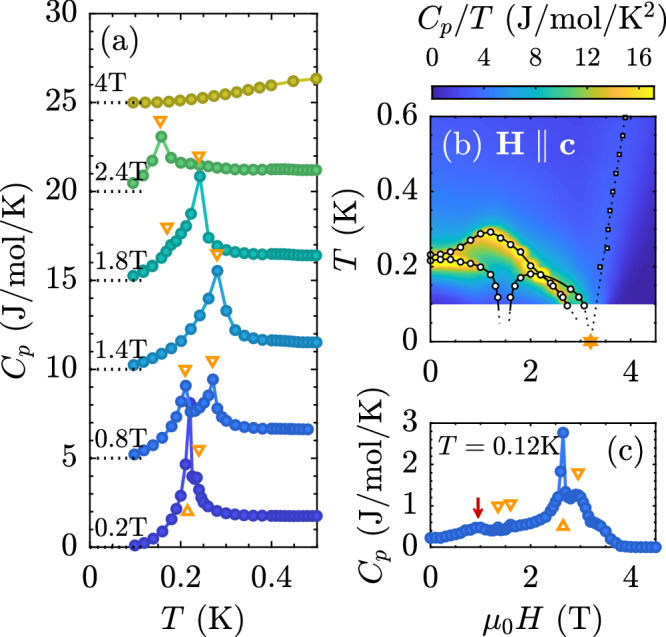
Specific heat of (CD_3_ND_3_)_2_NaRuCl_6_ in axial magnetic fields H∥c. **a** Exemplary *C*_*p*_(*T*) scans for various fixed fields *μ*_0_*H*. **b** Preliminary phase diagram. Color shows *C*_*p*_/*T* data, while circles identify sharp anomalies in heat capacity and squares denote the Schottky maximum above *H*_sat_. **c** Exemplary *C*_*p*_(*H*) scan at *T* = 0.12 K. Orange triangles highlight the peak positions in the data. Red arrow marks a broad maximum in *C*_*p*_ discussed in the text.

In an axial magnetic field **H**∥**c**, the two transitions at *T*_N1_ and *T*_N2_ begin to separate (see temperature scans in Fig. 5a). Magnetic order is enhanced by small applied fields, creating a reentrant bulge in the phase diagram with a maximal ordering temperature ${T}_{{\rm{N}}1}^{\max }\approx 0.3$ K. Meanwhile, *T*_N2_ moves towards lower temperatures and becomes suppressed, disappearing completely around 1.3 T. A small anomaly re-emerges above 1.6 T and seems to meet the main transition line at a bicritical point near *μ*_0_*H*_bc_ ≈ 2.2 T. At higher fields, we observe an additional pre-saturation phase, evident in field scans such as Fig. 5c as two consecutive anomalies around 2.65 and 2.95 T. Finally, above 3.2 T, we enter the pseudospin polarized regime where specific heat becomes gapped. We summarize this in the preliminary phase diagram shown in Fig. 5b, reminiscent of the 2D Heisenberg TLAF^41–43^ except for the extra pre-saturation phase. The spin gap increases linearly with field [white squares, denoting the Schottky maximum positions in *C*_*p*_(*T*, *H*)]. A fit to ${T}_{\max }\propto {(H-{H}_{{\rm{sat}}})}^{z\nu }$ yields a saturation field *μ*_0_*H*_sat_ = 3.20(2) T and crossover exponent *zν* = 1.15(5) for the proximate quantum critical point.

### Magnetoelastic effects

**Fig. 6:**
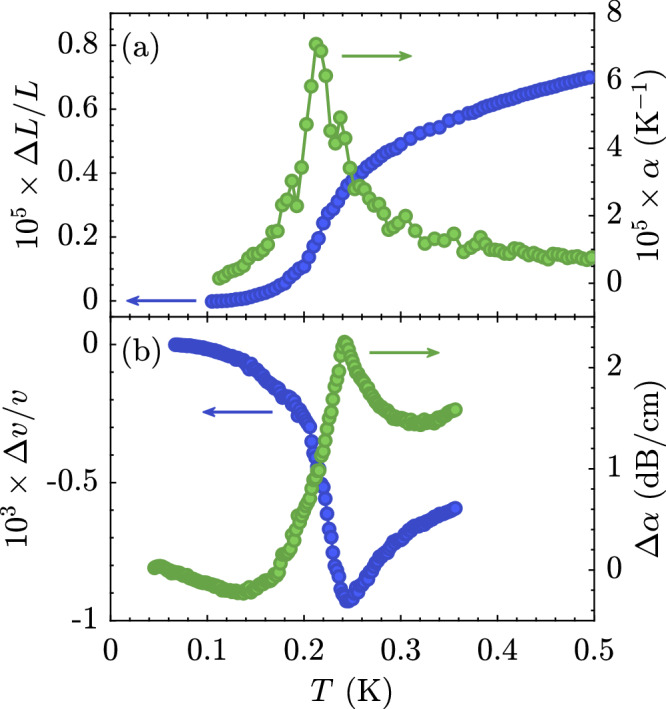
Magnetoelastic effects in (CD_3_ND_3_)_2_NaRuCl_6_. **a** Temperature-dependent sample dilation Δ*l*/*l* along the **a**^⋆^-axis, along with its numerical derivative, the thermal expansion coefficient $\alpha =\frac{1}{l}\frac{\partial \Delta l}{\partial T}$. **b** Relative changes in sound velocity and attenuation for the longitudinal *c*_11_-mode.

Given the spin-orbital entangled ground state doublet, the onset of magnetic order may have significant effects on the lattice (and vice versa). To probe such magneto-elastic coupling, we employ a combination of dilatometry and ultrasound measurements. In Fig. 6a, we show the relative change in sample length Δ*l*/*l* along **a**^⋆^ against temperature. The lattice contracts significantly below *T*_N_, which may be understood through an exchange-striction mechanism^44^: Exchange interactions depend on inter-atomic distances, so each bond acquires an extra stiffness contribution $\propto \langle \langle \hat{\mathbf{S}}_{i}\cdot \hat{\mathbf{S}}_{j}\rangle \rangle$ in the ordered state and the crystal shrinks to minimize the free energy (see Supplemental Material for details). Particularly interesting is the numerical derivative of this length change, i.e., the thermal expansion coefficient $\alpha (T)=\frac{1}{l}\frac{\partial \Delta l}{\partial T}$. Through Ehrenfest relations, the discontinuity in thermal expansion and heat capacity at *T*_N_ can be combined to estimate the initial pressure dependence of the transition temperature $\partial {T}_{{\rm{N}}}/\partial p={V}_{m}{T}_{{\rm{N}}}\frac{\Delta {\alpha }^{{\rm{crit}}}}{\Delta {C}_{p}^{{\rm{crit}}}}$. Here, Δ*α*^crit^ and $\Delta {C}_{p}^{{\rm{crit}}}$ represent the height of the critical anomaly in thermal expansion and heat capacity respectively, while *V*_*m*_ is the molar volume. We obtain a *huge* uniaxial pressure dependence ∂ln(*T*_N2_)/∂*p*_100_ ≈ 250%/GPa, indicating that the magnetic structure is highly sensitive to in-plane strains. This is on par with other readily pressure-tunable spin systems like TlCuCl_3_^45^ or *α*-RuCl_3_^46^. Analogous thermal expansion effects along the **c**-axis are an order of magnitude weaker (see Supplementary Fig. S7), consistent with a quasi-2D hierarchy of superexchange interactions.

Complementary information can be gleaned from ultrasound, where we measure relative changes in sound velocity Δ*v*/*v* and attenuation Δ*α* through the transition (see Fig. 6b). We focus on the longitudinal *c*_11_-mode, propagating in the triangular plane with a sound velocity *v*_11_ ≃ 2.1 km/s. Both probes exhibit strong anomalies at *T*_N_: Whereas the phonon frequencies soften near the onset of magnetic correlations (similar to Kohn anomaly), the attenuation—like all transport coefficients^47^—diverges at the transition, as sound waves become damped by critical fluctuations. Assuming an exchange-striction framework, we can estimate the magnetoelastic coupling constant ${g}_{\Gamma }\equiv \frac{\partial J}{\partial {\varepsilon }_{\Gamma }}$ for a particular strain component *ε*_*Γ*_. For the A_1_ “breathing” distortion of the triangular plane that we probe experimentally, we obtain *g*_A1_ ~ 40 K (see Supplementary Table T5). This is an order of magnitude larger than in similar triangular lattice systems like Ba_3_CoSb_2_O_9_^48^ or Cs_2_CuCl_4_^49,50^, signaling a strong coupling between pseudospin and lattice degrees of freedom.

**Fig. 7:**
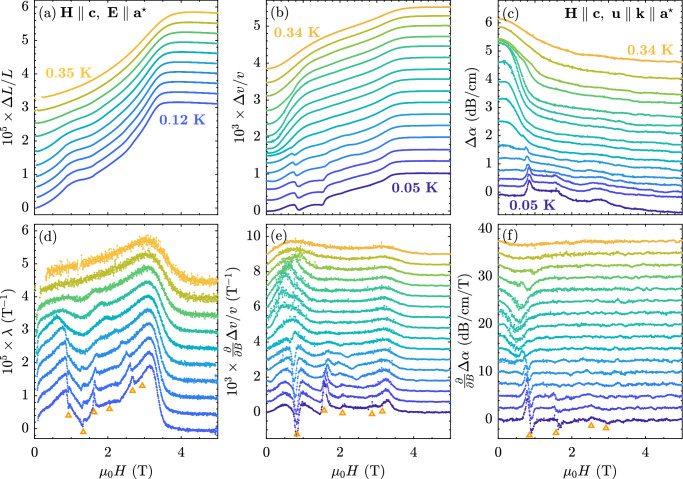
Field-induced magnetoelastic changes (top panels) and their numerical derivatives (bottom) for **H∥c** configuration. We show magnetostrictive changes in sample dilation (**a, d**), sound velocity (**b, e**) and attenuation (**c, f**) in the triangular plane. Various transition fields and measurement temperatures are indicated in the figure. Curves at different temperatures are offset for visibility.

This is borne out in additional field-induced measurements summarized in Fig. 7, revealing a much richer phase evolution than anticipated from heat capacity. The most pronounced anomalies appear in the region between 0.9 T and 1.6 T, where the sample length exhibits a plateau-like feature, while the sound velocity and attenuation show clear dips and peaks, respectively. The first of these features neatly matches a broad maximum observed in *C*_*p*_(*H*) around 0.9 T [red arrow in Fig. 5c]. Further anomalies become visible especially in the magnetostriction coefficient $\lambda (H)=\frac{1}{{\mu }_{0}l}\frac{\partial \Delta l}{\partial H}$ (Fig. 7d) or the analogous numerical derivatives of *Δv*/*v* and Δ*α*, revealing a cascade of field-induced phase transitions. In the low-temperature limit, there are six phases clearly identified by several probes, with transition fields near 0.9, 1.35, 1.6, 2.1, 2.6 and 3.2 T. With increasing temperature, all features gradually become broadened and disappear, leaving behind only paramagnetic behavior above ${T}_{{\rm{N}}1}^{\max }\approx 0.3$ K.

**Fig. 8:**
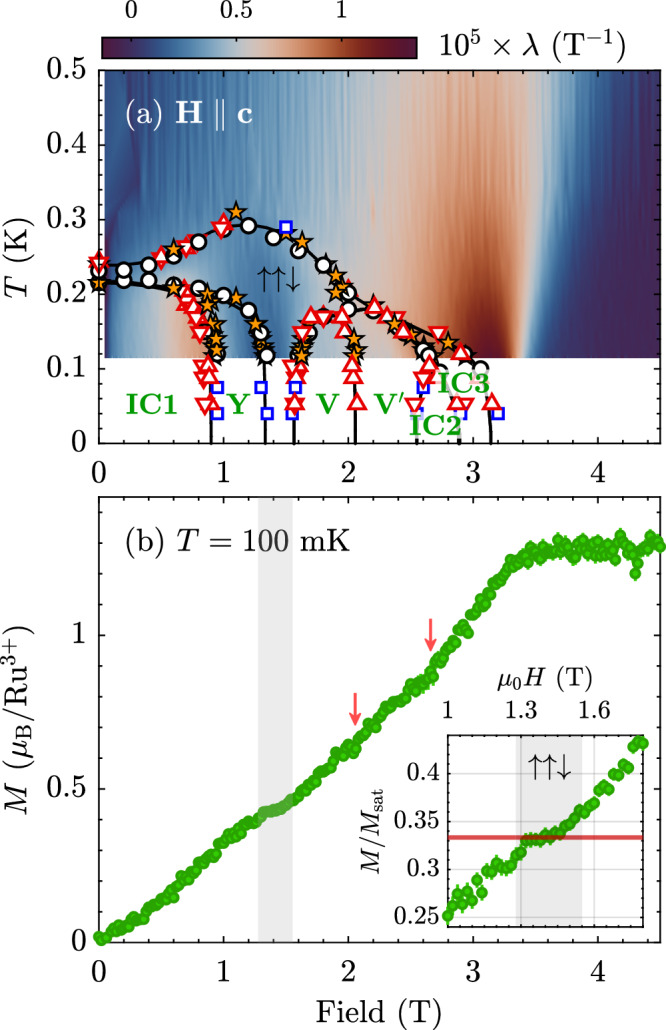
Phase diagram and magnetization curve of (CD_3_ND_3_)_2_NaRuCl_6_. **a** *H − T* magnetic phase diagram for the **H**∥**c** field direction. Color indicates the magnetostriction coefficient *λ*(*H*). White circles represent lambda anomalies from heat capacity. Orange stars are transitions extracted from magnetostriction. Red triangles (up/down facing) indicate anomalies in sound velocity and attenuation. Blue squares are transitions seen in neutron diffraction. **b** Magnetization curve along **H**∥**c** at *T* = 100 mK, measured with the Faraday balance technique. The inset shows a zoom-in on the “*↑↑↓*”-phase, displaying a plateau at one third of the saturation magnetization. Additional kinks matching the phase boundaries in (**a**) are marked by red arrows.

By combining the anomalies from all probes described above, we obtain the *H* − *T* phase diagram depicted in Fig. 8a. Note the presence of another slim presaturation phase between 2.6 and 2.9 T, stable only below *T* ~ 80 mK. The latter is clearly identified only by neutron diffraction (see below), though our ultrasound data do show some subtle features in this regime.

### Magnetization plateau

While the sequence of phases in Fig. 8a looks relatively complicated, there is an obvious starting point for understanding the magnetic behavior: The third phase, labeled “*↑↑↓*”. Its reentrant shape in the center of the *H* − *T* phase diagram and the second order transition line directly into the paramagnetic state are both typical features of the *m* = 1/3 magnetization plateau expected to emerge in an ideal Heisenberg triangular lattice system^41–43^. Being a collinear state, it is most strongly preferred by order-by-disorder and is therefore relatively stable with respect to most Hamiltonian perturbations. To confirm this, we carried out magnetization measurements at *T* = 100 mK using a home-made Faraday balance setup^51^, shown in Fig. 8b. Indeed, between 1.3 and 1.55 T, we observe a weak but distinctive kink in the magnetization curve, which is otherwise mostly convex up to saturation. In this regime, the magnetization reaches exactly one third of its saturation value *M*_sat_ = 1.29(1)*μ*_B_/Ru^3+^, corroborating our identification as a *m* = 1/3 magnetization plateau. Interestingly, the magnetostriction Δ*l*(*H*)/*l* (see Fig. 7a) seems to mimic the magnetization curve in the entire field range. This gives credence to the exchange-striction picture discussed above, in which the sample dilation would simply mimic the magnetic bond energies along the probing direction, i.e., $\Delta l/l \sim \frac{{g}_{{\rm{A}}1}}{k}{\sum }_{\langle ij\rangle \parallel {{\bf{a}}}^{\star }}\langle \langle \hat{\mathbf{S}}_{i}\cdot \hat{\mathbf{S}}_{j}\rangle \rangle$ where *k* is a stiffness constant. Note also the presence of weaker kink features around 2.05 and 2.6 T, matching the critical fields separating the V/V’ and V’/IC3 phases, respectively. Finally, we remark on the cusp in magnetization at *H*_sat_: The latter appears to be rounded significantly more than the 2*k*_B_*T* ~ 0.1 T expected from thermal broadening. Such behavior is typical of broken *SU*(2) symmetry in spin-orbit coupled systems, perhaps hinting at the presence of some non-trivial anisotropy terms^52^.

### Neutron diffraction

**Fig. 9:**
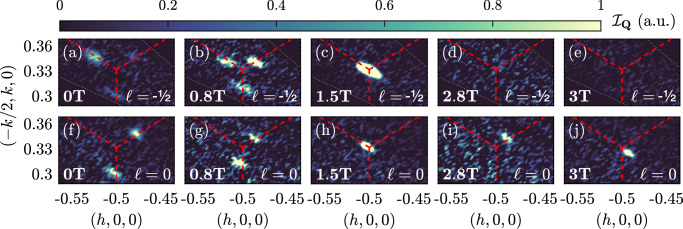
Magnetic neutron diffraction in (CD_3_ND_3_)_2_NaRuCl_6_ for longitudinal fields **H∥c**. Elastic scattering intensity at *T* ≲ 50 mK near the H-point **Q** ≈ (−2/3, 1/3, −1/2) (**a**–**e**) and K-point **Q** ≈ (−2/3, 1/3, 0) (**f**–**j**) for various magnetic field strengths. A paramagnetic background taken at *T* = 0.5 K has been subtracted point-by-point from all panels. Red dashed lines indicate the Brillouin zone boundaries and white dotted lines in (**a**–**e**) denote the detector edges.

**Fig. 10:**
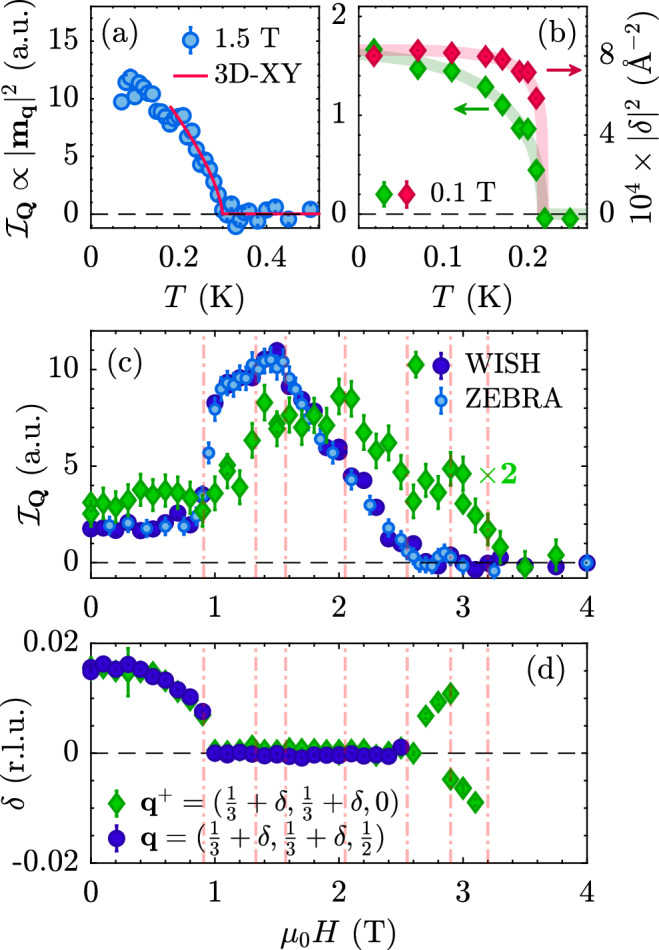
Temperature and field evolution of magnetic order in (CD_3_ND_3_)_2_NaRuCl_6_ for longitudinal fields **H∥c**. **a** Temperature dependence of the integrated intensity from the **Q** = (−2/3, 1/3, 1/2) reflection, taken in the plateau phase at *μ*_0_*H* = 1.5 T. **b** Evolution of the magnetic Bragg intensity and incommensurability of the **q**^+^ = (1/3 + *δ*, 1/3 + *δ*, 0) harmonics against temperature in the IC1 phase at *μ*_0_*H* = 0.1 T. Note the difference in curvature, showing that ∣**m**_**q**_∣∝̸∣*δ*∣. **c**, **d** Field dependence of the magnetic Bragg intensity and incommensurability *δ*(*H*) for both types of ordering vectors **q** and **q**^+^. Vertical lines denote the transition fields from Fig. 8a.

We carried out single-crystal neutron diffraction experiments to investigate the magnetic order realized across the entire phase diagram in **H**∥**c** orientation, the results of which are summarized in Figs. 9 and 10. As above, we start from the simplest phase, the collinear magnetization plateau. At *μ*_0_*H* = 1.5 T, we observe sharp magnetic Bragg peaks with a power-law onset of the order parameter below *T*_N_ ~ 0.29 K (see Fig. 10a). This is broadly consistent with the 2*β* ≈ 0.7 exponent expected for 3D XY universality - same class as the three-state Potts model in three dimensions, governing collinear *↑↑↓* order with a spontaneous choice of the “*↓*”-sublattice^53^. The propagation vector is **q** = (1/3, 1/3, 1/2), pointing to an antiferromagnetic nearest neighbor inter-plane coupling *J*_*c*_ > 0 (Fig. 9c). Additional reflections are visible in the *l* = 0 plane, corresponding to the second harmonic 2**q** ≡ (1/3, 1/3, 0) (Fig. 9h). Due to the non-bipartite three-sublattice order in each triangular layer, the inter-plane coupling is frustrated by the field. The Zeeman term prefers to cant *all* layers uniformly towards **H**∥**c**, leaving one in every three *J*_*c*_-bonds unsatisfied. Our magnetization plateau comprises alternating layers of “*↑↑↓*” and “*↑↓↑*” order (see Fig. 12 and “Discussion”). This results in a mixed FM-AFM inter-layer stacking involving three Fourier components **k** = **q,** **k** = 2**q** and **k** = 0 (i.e., the uniform magnetization), all of which are necessary to construct a semi-classical equal moment structure.

Towards lower fields, there is a commensurate-incommensurate lock-in transition at *μ*_0_*H*_*c*_ ≈ 0.95 T. Below this point, the magnetic Bragg peaks split into sets of three incommensurate (IC) reflections, equally spaced around the H and K-points (Fig. 9a, b, f, g). They move along the edges of the Brillouin zone and may be indexed by a propagation vector **q** = (*q*_IC_, *q*_IC_, 1/2), where *q*_IC_ ≡ 1/3 + *δ* ≃ 0.35 r.l.u. and *δ* is a field and temperature dependent IC shift. The magnetic texture spans 20 chemical cells in the triangular plane, corresponding to *a*_m_ ~ 143 Å. Symmetry imposes three degenerate arms of the *k*-vector star

2 $$\left\{\begin{array}{ll}{{\bf{q}}}_{1} & =({q}_{\mathrm{IC}},{q}_{\mathrm{IC}},1/2)\\ {{\bf{q}}}_{2} & =({q}_{\mathrm{IC}},-2{q}_{\mathrm{IC}},1/2)\\ {{\bf{q}}}_{3} & =(-2{q}_{\mathrm{IC}},{q}_{\mathrm{IC}},1/2),\end{array}\right\}$$

resulting in triplets of incommensurate peaks that could indicate either a multi-**q** structure or a trivial population of three-fold degenerate domains.

As seen in Fig. 9f, g, we also observe a secondary modulation, associated with a propagation vector **q**^+^ = (*q*_IC_, *q*_IC_, 0). Both ordering vectors adopt the exact same IC shift *δ*(*H*, *T*) (Fig. 10d), clearly indicating that these are Fourier components present in the same magnetic phase. We note that for finite *δ*, the *l* = 0 reflections are distinct from the second harmonics seen in the commensurate phases, i.e. ${{\bf{q}}}_{i}^{+}\not\equiv 2{{\bf{q}}}_{i}$ where *i* ∈ {1, 2, 3} enumerates the arms of the star. Instead, these peaks may be indexed as coupled harmonics of the form ${{\bf{q}}}_{i}^{+}\equiv {{\bf{q}}}_{j}+{{\bf{q}}}_{k}$. They represent direct interference terms between *different* arms of the propagation vector star, which cannot arise from separate magnetic domains. Therefore, the ground state must be a multi-**q** structure. Figure 10b depicts the temperature dependence of the order parameter and the incommensurability in the IC1 phase. Note that these quantities are not proportional to one another. We detect no changes in scattering intensity upon employing a field cooling or zero-field cooling protocol.

In the high-field limit, we observe a similar C-IC transition. The commensurate *l* = 1/2 peaks seen in the *m* = 1/3 plateau gradually lose intensity and disappear completely above 2.6 T. This leaves only the *l* = 0 reflections which move away from the K-point, resulting in a long-period incommensurate structure with simple ferromagnetic inter-plane stacking (Fig. 9d, e, i, j). We clearly observe *two* distinct IC presaturation phases: First, the peaks shift towards the M points, then around 2.9 T the trend reverses and the ordering vector moves towards *Γ*. In these high-field phases, only one satellite remains around each commensurate position, indicating a simple single-**q** magnetic order. The complete field dependence of the integrated intensity and incommensurability for both ordering vectors **q** and **q**^+^ is shown in Fig. 10c, d, in good agreement with the phase boundaries extracted from thermodynamics.

### Inelastic neutron scattering

**Fig. 11:**
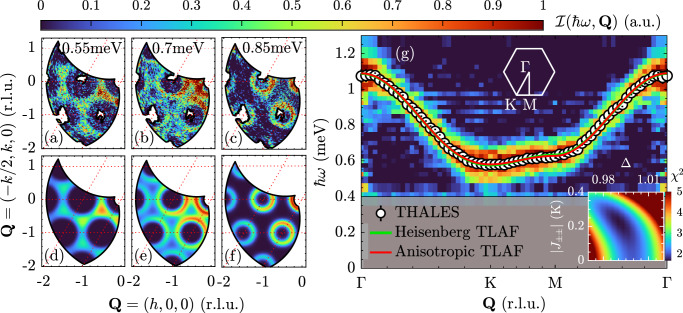
Inelastic neutron scattering spectra of (CD_3_ND_3_)_2_NaRuCl_6_ in the fully polarized state at *μ*_0_*H* = 7 T. **a**–**c** False color plots of INS intensity for 2D slices in momentum space taken at various constant energies. The white regions mask areas affected by Currat-Axe spurious scattering. **d**–**f** Simulated LSWT intensity in the same range using Eq. (3), convoluted with experimental resolution and form factor. **g** Dispersion relation along high symmetry directions. Markers show extracted peak positions, green/red lines are fits to a 2D Heisenberg/anisotropic model discussed in the text. The white hexagon represents a sketch of the Brillouin zone, where *Γ*, K and M label the high symmetry points in reciprocal space. Inset shows the *χ*^2^ loss function for anisotropic exchange parameters in the fit to Eq. (3).

In Fig. 11, we present the magnetic excitation spectrum of (CD_3_ND_3_)_2_NaRuCl_6_ in the triangular (*hk*0) plane, obtained from single-crystal neutron spectroscopy in the **H**∥**c** field-polarized regime at *μ*_0_*H* = 7 T. As expected, there is one resolution-limited spin-wave branch with a bandwidth 9*JS* ≈ 0.50 meV. The spin gap Δ = 0.58(1) meV to the lowest excitation at the K-point is in excellent agreement with the value Δ = *g*_*c*_*μ*_B_*μ*_0_(*H* − *H*_sat_) = 0.591(8) meV expected from ESR and thermodynamics.

Spin-wave theory is exact for the fully polarized ferromagnet^54^, so a fit to the extracted dispersion relation *ℏω*_**Q**_ may be used to determine the leading terms in the low-energy effective spin Hamiltonian. Since the *g*-factor and external field are known to high precision, we keep the Zeeman term fixed throughout this procedure. The data are well described by a simple nearest neighbor Heisenberg triangular lattice model with *J* = 1.32(1) K (Fig. 11g, green line). Potential further neighbor interactions *J*_2_ or *J*_3_ do not improve the fit and can be excluded at the ~2% level. We cannot probe the inter-plane couplings directly due to the scattering geometry (*hk*0) in this experiment. However, the classical saturation field *μ*_0_*H*_sat_ = 9*JS*/*g*_*c*_*μ*_B_ = 3.29(4) T nicely matches the *μ*_0_*H*_sat_ = 3.20(5) T observed experimentally. This places strong constraints on the leading AFM inter-plane coupling *J*_*c*_. We estimate *J*_*c*_ ≲ 0.1 *J*, clearly pointing to a quasi-2D system.

Given the spin-orbital entangled wavefunctions, we also attempt to fit a more general anisotropic Hamiltonian

3 $$\begin{array}{l}\hat{{\mathcal{H}}}=\mathop{\sum }\limits_{\langle ij\rangle }J({S}_{i}^{x}{S}_{j}^{x}+{S}_{i}^{y}{S}_{j}^{y}+\Delta {S}_{i}^{z}{S}_{j}^{z})\\\qquad+2{J}_{\pm \pm }[({S}_{i}^{x}{S}_{j}^{x}-{S}_{i}^{y}{S}_{j}^{y}){c}_{\alpha }-({S}_{i}^{x}{S}_{j}^{y}+{S}_{i}^{y}{S}_{j}^{x}){s}_{\alpha }]\\\qquad +{J}_{z\pm }[({S}_{i}^{y}{S}_{j}^{z}+{S}_{i}^{z}{S}_{j}^{y}){c}_{\alpha }-({S}_{i}^{x}{S}_{j}^{z}+{S}_{i}^{z}{S}_{j}^{x}){s}_{\alpha }].\end{array}$$

where $c{(s)}_{\alpha }=\cos (\sin ){\varphi }_{\alpha }$. Here the phases *φ*_*α*_ ∈ (0, 2*π*/3, 4*π*/3) associated with the translation vectors **a, b, a + b** give rise to a bond dependence on the pseudo-dipolar couplings *J*_±±_ and *J*_*z*±_. In practice, we are not sensitive to *J*_*z*±_, as it only enters the dispersion relation for a finite in-plane magnetic moment. This model results in a marginally better fit (*χ*^2^ ≈ 1.7 vs. *χ*^2^ ≈ 2), with *J* = 1.31(2) K, Δ = 1.00(2) and $| {J}_{\pm \pm }| =0.3{1}_{-0.23}^{+0.12}$ K. The system is clearly Heisenberg-like, with no signs of XXZ anisotropy. Our data do favor a strong *J*_±±_ term but with significant uncertainties, leaving neither one of the bond dependent terms well constrained. We leave this issue to future investigations in a transverse-field geometry^10^. A direct comparison between experimental data and simulated spin-wave intensities is shown in Fig. 11a–f through representative constant-energy cuts. Given the already excellent fit and limited sensitivity to these anisotropic terms, extending this model (e.g., with further neighbor interactions and their anisotropies) would risk overparameterization.

## Discussion

The novel quantum material (CD_3_ND_3_)_2_NaRuCl_6_ exhibits a perfect triangular lattice of Ru^3+^ ions realizing the spin-orbital entangled *j*_eff_ = 1/2 state. Perhaps its most striking property is the highly complex phase diagram in **H**∥**c** composed of seven distinct spin states, three of which are incommensurate with the lattice. The spin Hamiltonian is described at leading order by a purely 2D isotropic Heisenberg model with nearest neighbor coupling *J* ≈ 1.3 K. However, in the following we argue for three important perturbations to this picture, which drive the physics, resulting in the rich sequence of field-induced transitions: inter-plane coupling, exchange anisotropy, and magnetoelastic effects.

**Fig. 12:**
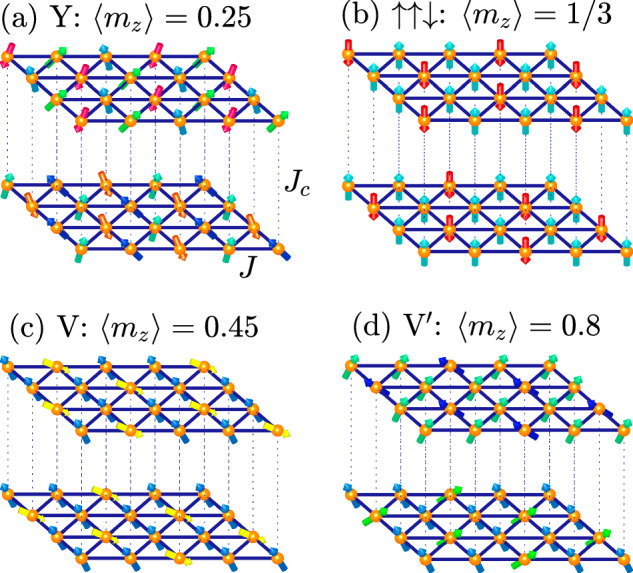
Field induced magnetic structures in (CD_3_ND_3_)2NaRuCl_6_. Sketch of the proposed spin configurations in the four commensurate phases **a** Y, **b** *↑↑↓*, **c** V and **d** ${{\rm{V}}}^{{\prime} }$.

At least the four commensurate states in the center of the phase diagram may be understood semi-classically by considering only the first of these terms, the inter-plane exchange. (CD_3_ND_3_)_2_NaRuCl_6_ realizes a direct AA-type stacking of triangular planes with an AFM nearest neighbor coupling *J*_*c*_ ≲ 0.1 *J*. Its competition with the magnetic field *H* results in a mixed FM-AFM inter-layer pattern which contains both **q** = (1/3, 1/3, 1/2) and 2**q** Fourier components. In Fig. 12, we sketch the proposed spin configurations in these phases, obtained through classical energy minimization of all possible six-sublattice groundstates with real-space perturbation corrections^55^ to model the effects of quantum order-by-disorder (for details, we refer the reader to Supplemental Fig. S10). The simplest is the *m* = 1/3 magnetization plateau, a collinear state forming alternating layers of *↑↑↓* and *↑↓↑* moments. This is followed by the well-known “Y” and “V” phases at slightly lower/higher fields, where the finite *J*_*c*_ results in an AFM canting of the in-plane spin components. Finally, a fourth phase is generally expected to arise in AA-stacked triangular lattice magnets with *J*_*c*_ > 0, often labeled “${{\rm{V}}}^{{\prime} }$”^56,57^. It is a simple consequence of the *J*_*c*_ vs. *H* competition, which favors a different inter-plane stacking close to the plateau and near saturation: In the former case the choice of minority spin is staggered between layers, whereas in the latter it is not. A first-order transition between these states is predicted at *H*_c_ ≈ 0.7*H*_sat_ in the Heisenberg limit^56^, only weakly depending on *J*_*c*_. This is in good agreement with the *H*_c_ ≈ 2.1 T (corresponding to ~ 0.65*H*_sat_) seen in experiment. An analogous transition has been identified in Ba_3_CoSb_2_O_9_^58,59^, the closest proxy to an ideal Heisenberg triangular lattice antiferromagnet known to date.

Let us turn to the IC behavior observed towards the edges of the phase diagram: The propagation vector **q** = (*q*_IC_, *q*_IC_, 1/2) cannot be explained by a Heisenberg model on a perfect triangular lattice and necessarily imposes some in-plane anisotropy term, either in spin-space or in real space. This anisotropy energy will tend to stabilize some complex non-coplanar order at all fields, but it has to compete with quantum order-by-disorder selection. The latter prefers more collinear/coplanar states due to their softer excitation spectrum, i.e., the commensurate Y, *↑↑↓* and V phases. Order-by-disorder corrections are strongest towards the center of the phase diagram (particularly around ~*H*_sat_/3), but completely disappear for the fully polarized ferromagnet as *H* → *H*_sat_. This provides a natural mechanism for IC order to develop, both in zero-field and just below saturation.

The simplest way to explain *q*_IC_ would be a structural transition, giving rise to a “static” $J-{J}^{{\prime} }$ isosceles distortion of the triangular plane that discriminates between chain-like *J* and zig-zag ${J}^{{\prime} }$ interactions. This model has been discussed thoroughly in the literature^60–62^, mostly in the context of the distorted triangular lattice magnets Cs_2_CuCl_4_^63,64^, Cs_2_CuBr_4_^65,66^ or Ca_3_ReO_5_Cl_2_^67,68^. But a “clean” experimental realization in the limit $({J}^{{\prime} }-J)\ll J$ has not been reported to date. The classical energy is minimized by a propagation vector ${q}_{{\rm{IC}}}=\arccos (-J^{\prime} /2J)/2\pi$, resulting in an IC spiral groundstate with three degenerate structural domains. After accounting for quantum renormalization, the detected *q*_IC_ ≈ 0.35 r.l.u. would correspond to an exchange ratio of $J^{\prime} /J\approx 1.06$^69–71^. The expected *H* − *T* phase diagram^60–62^ looks strikingly similar to experiment, with IC states towards *H* → {0, *H*_sat_} and the typical commensurate Y, *↑↑↓* and V phases in the center, where order-by-disorder selection dominates.

However, this option is ruled out by experiment: (CD_3_ND_3_)_2_NaRuCl_6_ *does* go through a structural transition at *T*^⋆^ ≈ 118 K, but our single-crystal x-ray diffraction data clearly establish that three-fold symmetry (i.e. $J^{\prime} =J$) remains intact, at least down to *T*_N_ (see Supplementary Material). Instead, we point out two alternative mechanisms which could account for the IC behavior, both of which rely on the strong spin-orbit effects.

An obvious culprit would be the magnetoelastic coupling. The latter could drive a magneto-structural phase transition at *T*_N2_, resulting in a “dynamic” $J-{J}^{{\prime} }$ isosceles distortion depending on both field and temperature. Such effects are often seen in pyrochlore systems like CdCr_2_O_4_ or HgCr_2_O_4_ (cf. “Spin-Peierls” effect^72,73^), where a *linear* gain in exchange energy upon distorting the lattice always leads to a small deformation below *T*_N_. As it turns out, this particular mechanism does not easily generalize to the 2D triangular lattice, since the leading correction to the classical energy $\Delta E\approx -\frac{9}{8}J{S}^{2}{\varepsilon }_{m}^{2}$ for a monoclinic lattice strain *ε*_*m*_ is only *quadratic* (see Supplementary Material). But analogous distortions may arise by invoking the pseudo Jahn-Teller effect in the presence of strong SOC, as recently proposed in 5*d*^5^ iridate perovskites to explain a small tetragonal distortion below *T*_N_^74^. Lattice deformations will modulate the spatial shape of the spin-orbital wavefunctions, which can generate new terms $\delta {{\mathcal{H}}}_{ij} \sim {g}_{\gamma }{\varepsilon }_{\gamma }{Q}_{ij}^{\gamma }$ in the Hamiltonian, describing the pseudospin-lattice coupling between two-site quadrupoles ${Q}_{ij}^{\gamma } \sim {\hat{S}}_{i}^{\alpha }{\hat{S}}_{j}^{\beta }$ of the appropriate symmetry and strains *ε*_*γ*_. An extension of this effect to the triangular lattice geometry would be an interesting problem to tackle theoretically.

However, there are several strong arguments to rule out any such magneto-elastic scenario. Firstly, if a lattice distortion is the primary driver for incommensurability, the $J-{J}^{{\prime} }$ anisotropy and the IC splitting of the **q**-vector must be directly proportional to the order parameter. This is in contradiction with our diffraction results. In the high-field “IC3” presaturation phase, *q*_IC_ moves *away* from the commensurate position even as the magnetic Bragg intensity goes to zero (see Fig. 10c, d). Furthermore, the temperature evolution of the order parameter ∣**m**_**q**_∣ and the IC shift *δ* in the IC1 phase exhibit a completely different curvature (see Fig. 10(b), clearly proving that the IC phases are not induced by magneto-elastic coupling. Another important clue comes from the low-field **q**^+^ satellites around the K-points (see Fig. 9f, g). Their presence as cross harmonics, coupling different arms of the propagation vector star, is direct proof that the IC1 phase exhibits multi-**q** order. This cannot be reconciled with a lattice distortion, which may only be stabilized through the energy gained by *breaking* three-fold symmetry. Finally, we have confirmed for all reflections in the family **Q** = (1/3, 1/3, 0) + ***δ***, that those with IC shift ***δ***∥**Q** - that is **Q** ≈ (0.35, 0.35, 0) and six-fold equivalents—carry no intensity, indicating an effect of the neutron polarization factor. This is inconsistent with all coplanar spiral configurations, favoring instead a multi-**q** order incorporating several longitudinal spin density wave contributions.

Bond-dependent exchange anisotropy presents a simple alternative to stabilize an incommensurate structure. Specifically, the Heisenberg-Kitaev model on a triangular lattice has been predicted to realize the so called ${{\mathbb{Z}}}_{2}$ vortex crystal^17,25,30^. Even for a tiny Kitaev-term ∣*K*∣ ≪ *J*, the Fourier transformed exchange matrix is minimized by three different ordering vectors

4 $${{\bf{q}}}^{\gamma }=\frac{{\hat{{\bf{e}}}}^{\gamma }}{\pi }\arccos \left(-\frac{J}{2(J+K)} \right),$$

one for each spin component *γ* ∈ (*x*, *y*, *z*). Locally, the magnetic structure would still mimic the typical 120^∘^ order. But on larger length scales, one would see a superlattice of topological defects - ${{\mathbb{Z}}}_{2}$ vortices - which crystallize into a triangular arrangement (see Supplementary Fig. S11). This structure has three main **q**-vectors, preserving three-fold symmetry, but with many higher harmonics to retain a uniform moment size - in agreement with the observation of **q**^+^ coupled harmonics. Since the bond-dependent terms break all continuous symmetries, the groundstate also acquires a finite spin gap Δ^17^, seemingly consistent with our heat capacity measurements indicating Δ ~ 0.4*J*.

In practice, the presence of a small Kitaev term in (CD_3_ND_3_)_2_NaRuCl_6_ should not be surprising. Bond dependent exchange anisotropies in the parallel-edge superexchange geometry have been proposed by several authors^14,25,26^. Given the bond angles deviating from 90^∘^ and the significant trigonal distortion Δ/*λ* ≃ − 0.5, we expect such terms to be sub-leading compared to *J*^40^. However, the observed propagation vector *q*_IC_ ≈ 0.35 r.l.u. can be generated by a modest ferromagnetic Kitaev term *K* ~ − 0.1 *J*, which seems completely realistic. In fact, the contribution from *J*_±±_ alone obtained through INS in the polarized regime would be significantly larger, with *K* = −2*J*_±±_ amounting to $K/J\approx -0.{4}_{-0.2}^{+0.3}$. This makes (CD_3_ND_3_)_2_NaRuCl_6_ a leading candidate to realize the ${{\mathbb{Z}}}_{2}$ vortex crystal phase. Given the large uncertainties on *J*_±±_ and completely undetermined *J*_*z*±_, more work should be put towards constraining these bond anisotropies. We note that the stability of this phase against finite inter-plane coupling *J*_*c*_, anisotropies away from the Heisenberg-Kitaev limit ${J}_{z\pm }\ne 2\sqrt{2}{J}_{\pm \pm }$, or magnetic field *H* have not been investigated theoretically, likely due to the inherent complexity of the vortex crystal state. All of these problems would be of imminent interest in understanding the groundstate of (CD_3_ND_3_)_2_NaRuCl_6_.

Finally, we comment on the tunability of this new structural archetype MA_2_ARuX_6_. Powder samples of several different members in this family have been previously synthesized in ref. ^35^, confirming that at least the alkali A and halide X can easily be replaced. This already gives huge flexibility in tuning the magnetism of these exciting new quantum materials. Especially the hierarchy between the small perturbing terms like inter-plane interactions, bond anisotropies or magnetoelastic coupling may be easily modified by changing the anions participating in superexchange.

In conclusion, the new quantum antiferromagnet (CD_3_ND_3_)_2_NaRuCl_6_ poses a rare model system to combine the effects of strong spin-orbit coupling endemic to 4*d*/5*d* transition metals and the geometric frustration on a triangular lattice. Its magnetism is described by a quasi-2D nearest-neighbor Heisenberg Hamiltonian with the addition of inter-plane interactions, bond anisotropies and magnetoelastic coupling as small perturbing terms, begetting an extremely rich phase diagram including several incommenasurate states. Of particular interest is the multi-**q** ground state realized in zero magnetic field, posing a prime candidate to host the exotic ${{\mathbb{Z}}}_{2}$ vortex crystal phase. This calls for polarized diffraction studies to confirm the explicit spin configuration and spectroscopy experiments to investigate its dynamics. (CD_3_ND_3_)_2_NaRuCl_6_ constitutes the first member in a large family of 4*d*/5*d*-based quantum triangular lattice magnets, opening new pathways to study the interplay between geometric frustration and spin-orbit effects.

## Methods

### Sample synthesis and characterization

Single crystal samples of (CD_3_ND_3_)_2_NaRuCl_6_ were grown using a hydrothermal method analogous to^35^. We obtain dark red crystals with highly symmetric hexagonal faces and typical sample mass of order 10−200 mg. Fully deuterated (≥98% D) precursors were employed in the growth to limit the incoherent H-scattering in neutron experiments. The structure and sample quality of all experimental probes was confirmed using a Bruker APEX-II single-crystal x-ray diffractometer. Detailed refinements to characterize the low-temperature structural transition were carried out at the I19 synchrotron beamline (Diamond, UK). Datasets of 1–5 × 10^4^ reflections were collected at *λ* = 0.6889 Å on several ~50 μm single crystal samples between *T* = 150 and 30 K. The DIALS^75^ and shelx^76^ software suites were employed for the data processing and structure solution, respectively.

### Thermodynamics

Magnetic susceptibility measurements on a *m* = 20 mg single crystal were performed in a commercial magnetic property measurement system (MPMS) SQUID magnetometer in a *μ*_0_*H* = 0.1 T probing field. High-*T* magnetization curves up to *μ*_0_*H* = 7 T were collected in the same configuration. The magnetization data at dilution temperatures were obtained on a 2 mg sample using a home-built Faraday balance setup^51^ and calibrated to absolute units using the SQUID data at 2 K as reference. For the ESR experiments, a tunable-frequency spectrometer (similar to the one described in ref. ^77^) was employed, equipped with a 16 T superconducting magnet. VDI microwave-chain sources (product of Virginia Diodes, Inc., USA) were used to generate radiation in the frequency range of 50–360 GHz. A hot-electron n-InSb bolometer (product of QMC Instruments Ltd, UK), operated at 4.2 K, was employed as a THz detector. For the frequencies below 50 GHz a microwave vector network analyser (MVNA, production of AB Millimeter, France) was used. Sample holders in the Faraday and Voigt configuration were used for **H**∥**c** and **H**∥**a** measurements respectively. 2,2-diphenyl-1-picrylhydrazyl (DPPH) was used as a standard frequency-field marker. The heat capacity of a 0.4 mg single crystal sample was measured with the standard relaxation calorimetry technique on a 9 T Physical Property Measurement System (PPMS) with a ^3^He-^4^He dilution refrigerator insert. Magnetostriction data were taken in the same configuration using a *L* = 1.48 mm long single crystal with natural faces in the (100) and (001) cleavage planes. The sample dilation was determined with a miniature capacitive dilatometer^78^ in conjunction with an Andeen-Hagerling 2700A bridge, operated at 1.11 kHz. Sound velocity measurements of the longitudinal *c*_11_ mode (polarization **u** and propagation **k** along the **a**^⋆^-axis) were carried out at the Dresden High Magnetic Field Laboratory using a pulse-echo method with phase-sensitive detection^79,80^. LiNbO_3_ transducers were bonded with Thiokol to two parallel (100) faces of a *L* = 2.8 mm long single crystal sample. Experiments were carried out at *f* = 40 MHz using a ^3^He-^4^He dilution refrigerator equipped with an 18 T magnet.

### Neutron scattering

Single-crystal neutron diffraction experiments were performed at WISH (ISIS, UK)^81,82^ and ZEBRA (PSI, Switzerland) on the same *m* = 55 mg sample aligned in the (*hk*0) scattering plane. The crystal was mounted in a ^3^He-^4^He dilution refrigerator together with a 10 T (6 T) vertical cryomagnet. A constant wavelength *λ* = 1.383 Å (Ge monochromator) was chosen for the latter experiment. The incommensurate positions and intensities from WISH were extracted by fitting 2D Gaussian profiles with a fixed resolution (Δ*q*_∥_, Δ*q*_⊥_) to the data. These data were analyzed using the mantid software suite^83^.

Inelastic neutron scattering experiments were carried out on the cold triple-axis spectrometer THALES (ILL, France)^84^ with Flatcone multi-analyzer setup. 16 single crystals were coaligned in the *ab* scattering plane for a total sample mass of *m* ≃ 1.9 g. The probe was installed with (*hk*0) scattering geometry in a dilution refrigerator with 10 T vertical cryomagnet and cooled to *T* ~ 70 mK. All measurements were taken at a final energy *E*_*f*_ = 4.06 meV (FWHM resolution Δ*E* ≈ 175 *μ*eV), fixed by the Flatcone setup. 35 constant-energy slices were collected in the field-polarized regime at *μ*_0_*H* = 7 T, rotating 90^∘^ through the scattering plane in 0.5^∘^ steps. The triple-axis geometry allows for so called “Currat-Axe" spurions^85^, undesirable scattering events that occur when the sample angle accidentally matches a Bragg reflection, resulting in a spurious peak upon incoherent/thermal diffuse scattering in the analyzer. These features follow known paths in reciprocal space and are simply masked out (see white spots in Fig. 11a–c). To reconstruct the spin-wave dispersion relation, regions affected by Currat-Axe spurions are discarded, a constant background was subtracted, the data were normalized by form factor and averaged over >20 equivalent paths through several Brillouin zones. Further details about the sample and fitting procedures may be found in Supplementary Figs. S8 and 9. The SpinW software^86^ was employed for the spin-wave intensity calculations.

## Supplementary information

Supplementary Information

## Data Availability

All data are available upon reasonable request to the corresponding author. The neutron scattering data collected at WISH (STFC ISIS Neutron and Muon Source) and THALES (Institut Laue-Langevin) are available at 10.5286/ISIS.E.RB2420060 & 10.5286/ISIS.E.RB2520045 and 10.5291/ILL-DATA.4-01-1857, respectively.
