# Supplementary material for: ℤ2 vortex crystal candidate in the triangular S = 1/2 quantum antiferromagnet
Source: NPJ Quantum Mater. 2026 May 26;11(1):69. doi: 10.1038/s41535-026-00894-4 (PMC13485684; doi:10.1038/s41535-026-00894-4)
Supplement: Supplementary file 1 [file 41535_2026_894_MOESM1_ESM.pdf]

Supplemental Material for "$\mathbb{Z}_2$ Vortex Crystal Candidate in the Triangular $S = 1/2$ Quantum Antiferromagnet"

Jakob Nagl,[1,*] Kirill Yu. Povarov,[2] Benjamin Duncan,[1] Catharina Näppi,[1] Dmitry Khalyavin,[3] Pascal Manuel,[3] Fabio Orlandi,[3] Jeremy Sourd,[2] Beat Valentin Schwarze,[2] Freya Husstedt,[2,4] Sergei A. Zvyagin,[2] Oksana Zaharko,[5] Paul Steffens,[6] Arno Hiess,[6,7] David R. Allan,[8] Sarah A. Barnett,[8] Zewu Yan,[1] Severian Gvasaliya,[1] and Andrey Zheludev[1,†]

[1] *Laboratory for Solid State Physics, ETH Zürich, 8093 Zürich, Switzerland*
[2] *Dresden High Magnetic Field Laboratory (HLD-EMFL) and Würzburg-Dresden Cluster of Excellence ct.qmat, Helmholtz-Zentrum Dresden-Rossendorf (HZDR), 01328 Dresden, Germany*
[3] *ISIS Facility, Rutherford Appleton Laboratory, Chilton, Didcot, Oxon OX11 0QX, United Kingdom*
[4] *Institut für Festkörper- und Materialphysik, Technische Universität Dresden, 01062 Dresden, Germany*
[5] *PSI Center for Neutron and Muon Sciences, Forschungsstrasse 111, 5232 Villigen, PSI, Switzerland*
[6] *Institut Laue-Langevin, 71 avenue des Martyrs, CS 20156, 38042 Grenoble Cedex 9, France*
[7] *European Spallation Source ERIC, P.O. Box 176, 22100 Lund, Sweden*
[8] *Diamond Light Source, Harwell Science and Innovation Campus, Didcot, Oxfordshire OX11 0DE, UK*
(Dated: April 28, 2026)

In this supplement, we provide supporting information about the low-temperature crystal structure, the single-ion fitting procedures & ESR data, the magnetoelastic coupling, our inelastic neutron spectroscopy experiment and the proposed magnetic structures.

I. CRYSTAL STRUCTURE & SPIN HAMILONIAN

$(CD_3ND_3)_2NaRuCl_6$ adopts a trigonal $P\bar{3}m1$ structure at room temperature [1], realizing an ideal triangular lattice of Ru^{3+} ions in the spin-orbital entangled $j_{\text{eff}} = 1/2$ state. The local point symmetry at the magnetic site amounts to $.\bar{3}m$. In this case, the most general 2D spin Hamiltonian for nearest neighbors, including all anisotropy terms allowed by symmetry, contains *four* exchange parameters. In the global Cartesian frame ($\hat{x} \parallel a$, $\hat{z} \parallel c$) it may be written as

$$\mathcal{H}_{\parallel} = \sum_{\langle i,j\rangle} J(S_i^x S_j^x + S_i^y S_j^y + \Delta S_i^z S_j^z) \tag{1}$$
$$+2J_{\pm\pm}[(S_i^x S_j^x - S_i^y S_j^y)\cos(\varphi_\alpha) - (S_i^x S_j^y + S_i^y S_j^x)\sin(\varphi_\alpha)]$$
$$+J_{z\pm}[(S_i^y S_j^z + S_i^z S_j^y)\cos(\varphi_\alpha) - (S_i^x S_j^z + S_i^z S_j^x)\sin(\varphi_\alpha)].$$

Here, the phases $\varphi_\alpha \in (0, 2\pi/3, 4\pi/3)$ associated with the primitive translation vectors $\mathbf{a}, \mathbf{b}, \mathbf{a}+\mathbf{b}$ give rise to a bond dependence on the anisotropic $J_{\pm\pm}, J_{z\pm}$ couplings. These triangular layers exhibit a direct AA-stacking, resulting in a simple inter-plane coupling restricted to XXZ form

$$\mathcal{H}_{\perp} = \sum_{\langle i,j\rangle} J_c(S_i^x S_j^x + S_i^y S_j^y + \Delta_c S_i^z S_j^z). \tag{2}$$

In both cases, a superexchange between next-nearest neighbors would involve significantly longer bond lengths (12.37 Å vs. 7.14 Å in the triangular plane, 9.86 Å vs. 6.80 Å between layers) and is unlikely to play an important role.

Towards lower temperatures, $(CD_3ND_3)_2NaRuCl_6$ goes through a structural transition. This is best seen in additional heat capacity measurements depicted in Fig. S1, carried out on a 4.5 mg single crystal sample in a 9 T Physical Property Measurement System (PPMS). The data show a clear lambda anomaly at $T^\star \approx 118$ K, indicative of a continuous phase transition. An entropy release of order $\Delta S \sim R\log(2)$ would suggest a spontaneous choice between *two* structural configurations.

To characterize the structural changes near $T^\star$, we performed high-resolution single crystal x-ray experiments at the I19 synchrotron beamline (Diamond, UK). Shelx-based refinement results on a representative sample are summarized in Tables T1 to T3. A high-temperature dataset collected at 150 K ($T > T^\star$) yields excellent agreement to a model

* jnagl@ethz.ch
† zhelud@ethz.ch; http://www.neutron.ethz.ch/

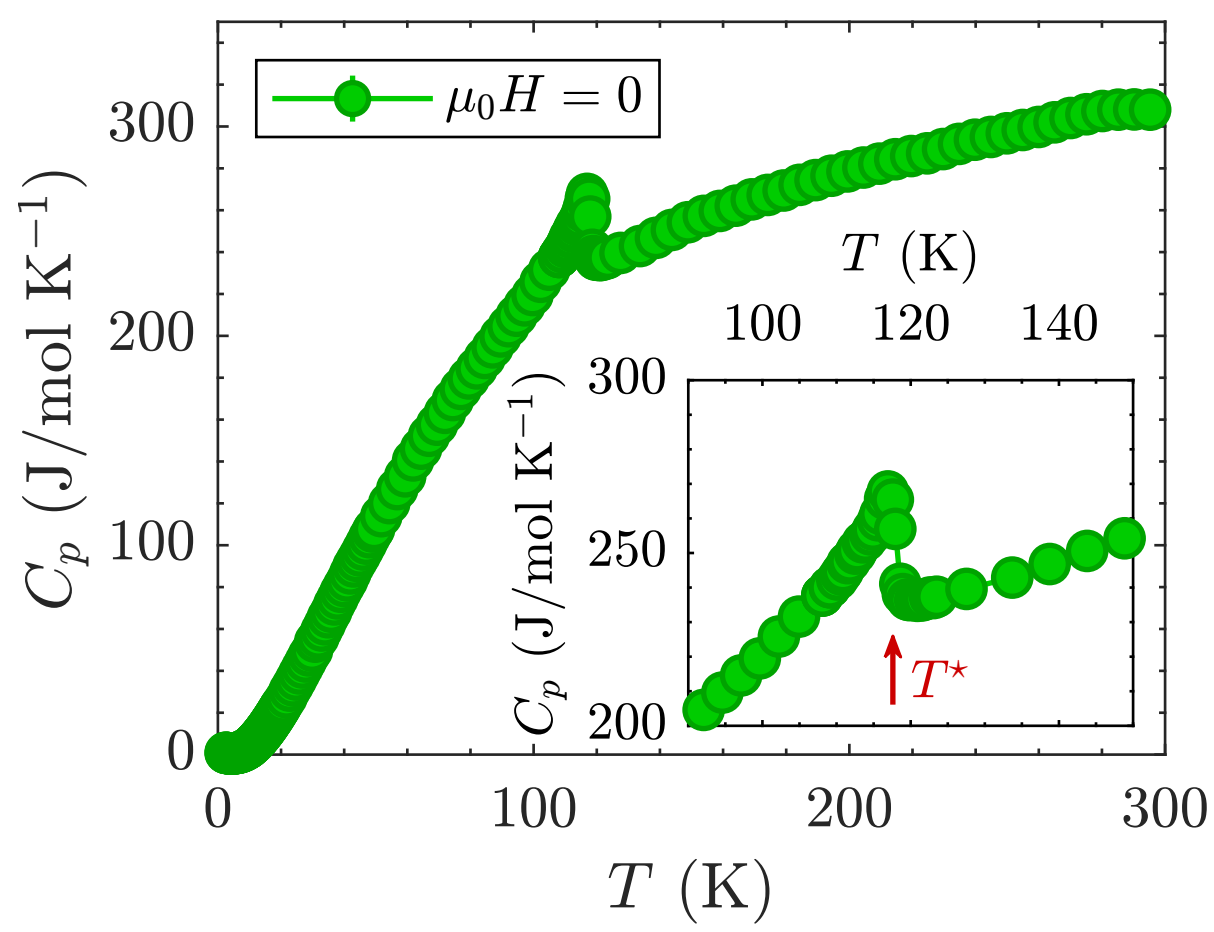

FIG. S1. Heat capacity in $(CD_3ND_3)_2NaRuCl_6$ against temperature, depicting a structural transition at $T^\star$. The inset shows a zoom-in close to the lambda anomaly.

TABLE T1. Summary of single crystal x-ray refinements of the chemical structure $(CD_3ND_3)_2NaRuCl_6$ at 150 K ($T > T^\star$) and 30 K ($T < T_\star$).

Characteristics	$T = 150$ K	$T = 30$ K, High Sym.	$T = 30$ K, Low Sym.
Chemical formula	$(CD_3ND_3)_2NaRuCl_6$	$(CD_3ND_3)_2NaRuCl_6$	$(CD_3ND_3)_2NaRuCl_6$
Wavelength (Å)	0.6889	0.6889	0.6889
Crystal System	Trigonal	Trigonal	Trigonal
Space Group	$P\bar{3}m1$	$P\bar{3}m1$	$P\bar{3}$
a, b (Å)	7.1964(1)	7.1435(1)	7.1435(1)
c (Å)	6.7939(1)	6.7984(1)	6.7984(1)
α, β (deg)	90	90	90
γ (deg)	120	120	120
V (Å^3)	304.71(1)	300.44(1)	300.44(1)
Z	1	1	1
ρ_{calc} (g/cm^3)	2.25	2.28	2.28
$2\theta_{\max}$ (deg)	116.74	116.74	116.74
Measured Reflections	37577	31016	31016
Independent Reflections	1750	1796	1796
Refined Parameters	18	18	23
R_1 (%)	1.33	9.68	2.45
wR_2 (%)	3.69	38.53	6.73
Goodness of Fit S	1.075	3.372	1.217
Twinning Ratio f	-	-	1.62 : 1
Diff. density (e/Å^3)	0.65 / -0.91	15.35 / -13.45	2.11 / -3.27

based on the nominal $P\bar{3}m1$ crystal structure, with $R_1 = 1.3\%$ and less than 1 e/Å^3 difference in electronic density. Due to their weak scattering, the light deuterium atoms are constrained with a riding model, assuming an ideal CH_3 group with variable distance and thermal parameters fixed by the non-riding atom. Repeating an analogous refinement on a 30 K ($T < T^\star$) dataset results in a significantly worse $R_1 = 9.68\%$, indicating some change in the chemical structure. None of the space groups consistent with the apparent Laue class $\bar{3}m1$ improve the fit.

This tension may be resolved by considering a transition into a low-symmetry $P\bar{3}$ structure. Such a model allows for 2-fold merohedral twinning, which can disguise the "true" Laue class and is consistent with the observed release of entropy. Here the mirror plane is lost, allowing the $RuCl_6$ octahedra and CD_3ND_3 molecules to rotate by $\pm\psi$ around the z-axis passing through the high-symmetry site ($1b$ and $2d$ respectively). Equivalently, this corresponds to a loss of 2-fold rotational symmetry around $\mathbf{a}$, $\mathbf{b}$ or $\mathbf{a} + \mathbf{b}$. A possible twinning matrix would be $[-1\ 0\ 0;\ 1\ 1\ 0;\ 0\ 0\ 1]$. The real (and reciprocal) space lattices of both twin domains overlap perfectly and the indexing of peaks does not change with respect to the parent structure. This model yields excellent agreement with the data, where $R_1 = 2.4\%$. Again, the deuterium positions are constrained by a riding model, though now the rotation angle of the CH_3 group also becomes a free parameter.

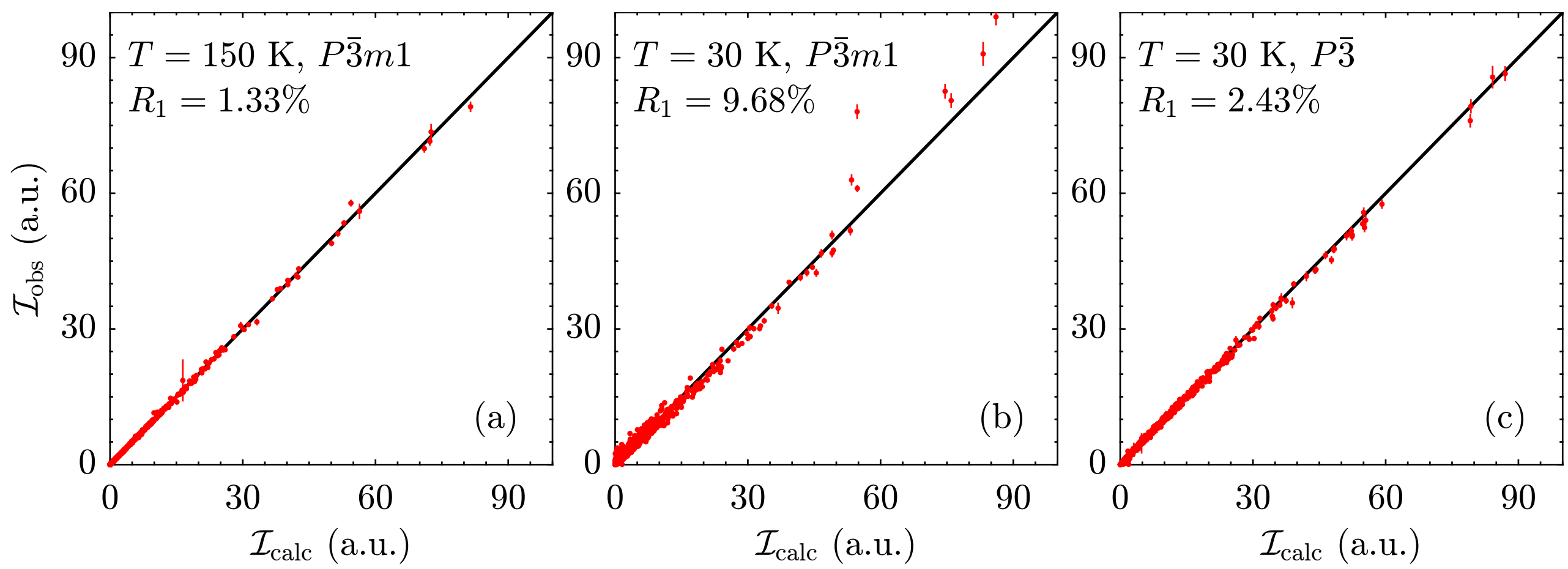

FIG. S2. Observed versus calculated x-ray intensities at 150 K in $P\bar{3}m1$ space group (a), at 30 K with the nominal $P\bar{3}m1$ structure (b) and at 30 K for a lower symmetry $P\bar{3}$ model including 2 merohedral twins. The solid line shows the 1:1 agreement in all panels.

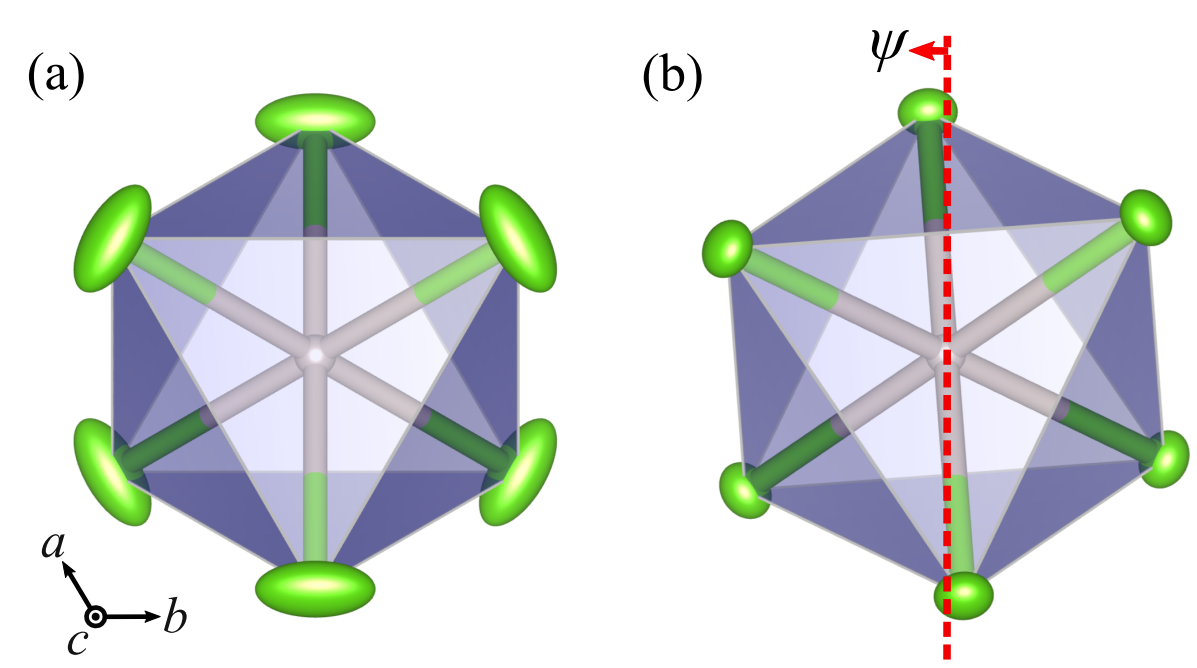

FIG. S3. $RuCl_6$ octahedra for single-crystal x-ray refinements at 30 K against different structural models. (a) Nominal $P\bar{3}m1$ structure and (b) lower-symmetry $P\bar{3}$ structure with parameters in tables T1 to T3. The green ellipsoids illustrate the anisotropic thermal displacements, while the red line in (b) emphasizes the rotation of $RuCl_6$ octahedra by ψ.

Final refinement results and structural differences between both models are visualized in Fig. S2 and Fig. S3. The rotation angle of the $RuCl_6$ octahedra at 30 K - controlling the degree of symmetry breaking - amounts to $\psi = \pm 2.95(1)^\circ$. Hints of the structural change are seen even in the high-symmetry $P\bar{3}m1$ model: The thermal parameters on chlorine are highly anisotropic, elongated in the triangular plane to cover both $\pm\psi$.

We note that a non-centrosymmetric space group $P3$ would also be consistent with the merohedral twinning scenario discussed above. However, our refinement attempts yield no improvement in R-factors despite the extra parameters. Analogous refinements were carried out on several single crystal samples, always pointing to the $P\bar{3}m1 \to P\bar{3}$ transition with analogous R-factors ($8-10\%$ vs. $1.5-2.5\%$ for $P\bar{3}m1$ and $P\bar{3}$ models respectively). Similar structural changes have been reported in the triangular lattice systems $RbFe(MoO_4)_2$ [2] and $Na_2BaCo(PO_4)_2$ [3, 4], making this a relatively common structural motif.

Let's discuss the potential impact of this structural phase transition on the magnetism. Anti-symmetric Dzyaloshinskii-Moriya terms remain forbidden by symmetry, at least down to $T_{\rm N}$ (the same goes for anything akin to a $J-J'$ isosceles distortion). However, the lower point symmetry $.\bar{3}$ at the Ru^{3+} site allows for two new symmetric anisotropy terms in the triangular lattice Hamiltonian of Eq. 1. We can parameterize these by adding separate phase shifts to the bond angles $\varphi_\alpha \to \varphi_\alpha + \delta\phi$, one for each of the bond-dependent terms $J_{\pm\pm}$ and $J_{z\pm}$.

As for the inter-plane coupling, the main effect of the transition would be to allow for two inequivalent diagonal terms $J_a \neq J_b$ [see Fig. S4(a)]. This has been explored previously in both $RbFe(MoO_4)_2$ [5, 6] and $Na_2BaCo(PO_4)_2$ [4]. The mean-field inter-plane exchange energy is given as

$$E(q_c) = -\frac{3}{2}JS^2 - \frac{1}{2}[3(J_a+J_b)-2J_c]S^2\cos(2\pi q_c) + \frac{3\sqrt{3}}{2}[J_b-J_b]S^2\sin(2\pi q_c) \quad (3)$$

per site. In practice, any $J_a \neq J_b$ will entail an incommensurate (IC) *inter*-plane stacking $q_c \neq 1/2$, incompatible with experiment. We solve Eq. 3 numerically and plot the resulting inter-plane propagation vector in Fig. S4(b). By

TABLE T2. Fractional atomic coordinates and thermal displacement parameters deduced from a refinement of single crystal x-ray diffraction data at $T = 150$ K against the nominal structural model with space group $P\bar{3}m1$ (No. 164). The light deuterium atoms are constrained with a riding model (CH_3 group with variable distance).

Atom	Wyckoff	x	y	z	Equiv. B_{iso}
Na	1a	0	0	0	1.596(7)
Ru	1b	0	0	0.5	0.814(2)
Cl	6i	0.69054(2)	0.84527(2)	0.29605(2)	2.002(2)
N	2d	0.33333	0.66667	0.6592(1)	2.09(1)
C	2d	0.33333	0.66667	0.8741(2)	2.32(2)
D(1)	6i	0.26714	0.73811	0.61452	3.13
D(2)	6i	0.40828	0.59336	0.92220	3.48

B_{11}	B_{22}	B_{33}	B_{23}	B_{31}	B_{12}
1.95(1)	1.95(1)	0.89(1)	0	0	0.974(6)
0.875(2)	0.875(2)	0.692(2)	0	0	0.437(1)
1.077(2)	2.002(3)	1.123(3)	-0.114(1)	-0.227(2)	0.539(2)
2.487(2)	2.487(2)	1.287(2)	0	0	1.24(1)
2.835(2)	2.835(2)	1.303(2)	0	0	1.42(1)

TABLE T3. Same as Table T2, but for a refinement of the $T = 30$ K data against the reduced-symmetry space group $P\bar{3}$ (No. 147). Here the mirror plane is lost, allowing the Cl octahedra and CD_3ND_3 molecules to rotate. The light deuterium atoms are constrained with a riding model (CH_3 group with variable distance & rotation angle).

Atom	Wyckoff	x	y	z	Equiv. B_{iso}
Na	1a	0	0	0	0.70(1)
Ru	1b	0	0	0.5	0.345(2)
Cl	6g	0.68921(4)	0.82456(4)	0.29564(3)	0.575(3)
N	2d	0.33333	0.66667	0.3401(2)	0.86(1)
C	2d	0.33333	0.66667	0.1222(2)	0.99(2))
D(1)	6g	0.22734	0.69112	0.61527	1.26
D(2)	6g	0.44432	0.63561	0.92585	1.50

B_{11}	B_{22}	B_{33}	B_{23}	B_{31}	B_{12}
0.821(2)	0.821(2)	0.474(2)	0	0	0.411(9)
0.341(2)	0.341(2)	0.353(3)	0	0	0.166(2)
0.473(6)	0.644(7)	0.531(6)	-0.038(4)	-0.066(4)	0.220(5)
0.963(2)	0.963(2)	0.655(3)	0	0	0.48(1)
1.145(3)	1.145(3)	0.687(3)	0	0	0.57(2)

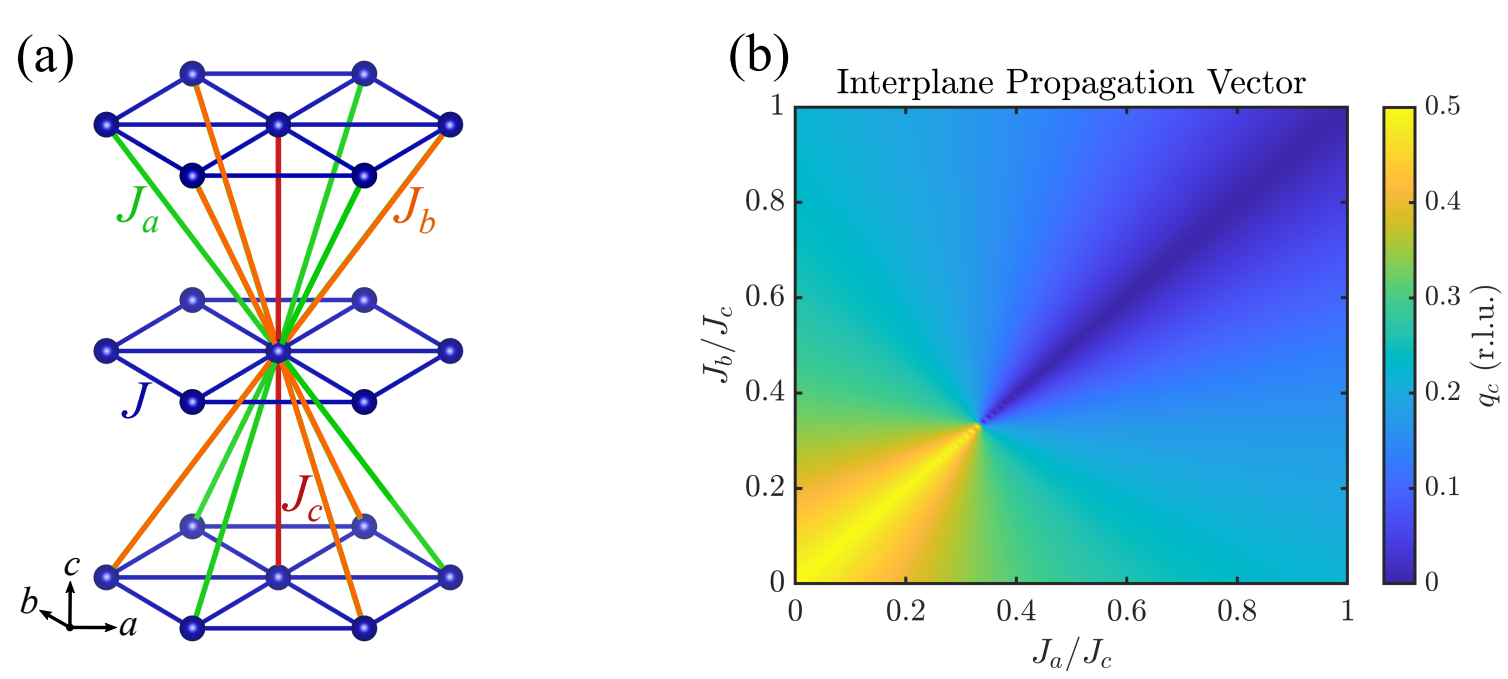

FIG. S4. Effects of structural transition on the inter-plane coupling. (a) The diagonal next-nearest neighbor interactions become inequivalent by symmetry, i.e. $J_a \neq J_b$. (b) Out-of-plane propagation vector component q_c obtained by minimizing the classical energy in Eq. 3. An $J_a \neq J_b$ should result in IC stacking along the c-axis.

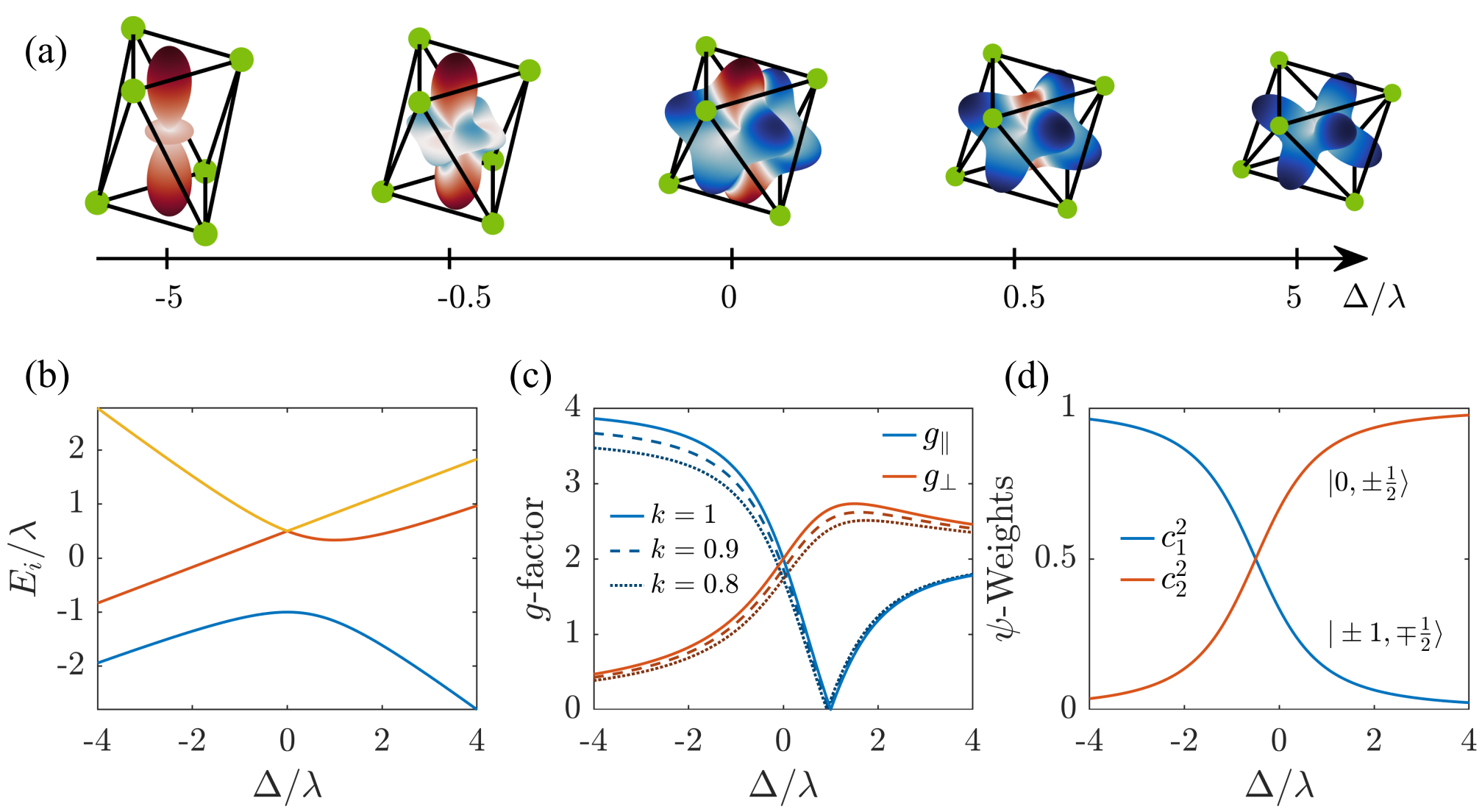

FIG. S5. Effects of trigonal distortion on the spin-orbital wavefunctions. (a) Sketch of the spatial shape of the pseudospin wavefunctions for different ratios Δ/λ. Red/blue color indicates spin up/down polarization of the hole. In (b,c,d) we show the dependence of the energy spectrum, g-factors and ground state weights on the trigonal distortion. Results of different k due to covalency effects are also depicted.

setting $q_c \equiv 1/2$ we can restrict $J_a \approx J_b < J_c/3$, so the main physics is captured purely by J_c.

Finally, we remark on our angular resolution in the XRD experiment. We have collected reflections at scattering angles up to $2\theta \approx 116°$, corresponding to an experimental resolution of order $\Delta d/d \sim 0.05\%$. We can compare this value to the monoclinic lattice distortion required to explain the zero-field incommensurate reflections in terms of a "static" $J - J'$ isosceles model. Given the scale of magnetoelastic coupling determined in Sec. IV, we estimate that a distortion at least of order $\gtrsim 0.1\%$ would be necessary, even after accounting for quantum renormalization of the classical ordering wavevector $q_{\rm IC} = \arccos(-J'/2J)/2\pi$. Therefore, such effects should be clearly resolved as peak splitting and can be ruled out by experiment. We note that structural changes even an order of magnitude smaller than the experimental resolution can typically be identified by a systematic increase in R-factors below $T^\star$ (not observed).

II. SINGLE-ION FITS & SPIN-ORBITAL WAVEFUNCTIONS

Here we summarize our fitting procedures of the single-ion + mean-field model discussed in the main text. We keep only the low-energy $\hat{\ell}_{\rm eff} = 1$ (orbital) and $\hat{S} = 1/2$ (spin) degrees of freedom, corresponding to a single hole in the t_{2g} manifold. This results in the on-site Hamiltonian

$$\mathcal{H} = \lambda \hat{\boldsymbol{\ell}} \cdot \hat{\mathbf{S}} + \Delta \hat{\ell}_z^2 - \mu_{\rm B} \mathbf{H} \cdot \hat{\mathbf{M}} \tag{4}$$

where $\hat{\mathbf{M}} = 2\hat{\mathbf{S}} - k\hat{\boldsymbol{\ell}}$ is the magnetization and $k \lesssim 1$ accounts for a small reduction in orbital moment due to hybridization with Cl^- anions (i.e. covalency effects [7, 8]). By diagonalizing $\mathcal{H}$, we obtain the energy spectrum E_i and eigenstates $|\psi_i\rangle$ (see Fig. S5). For dominant spin-orbit coupling $|\Delta/\lambda| \lesssim 1$, the ground state is a spin-orbital entangled $j_{\rm eff} = 1/2$ Kramers doublet, written as

$$|\pm\rangle = c_1 |\pm1, \mp1/2\rangle + c_2 |0, \pm1/2\rangle \tag{5}$$

$$\text{where} \qquad c_{1,2} = \pm\sqrt{\frac{1}{2} \mp \frac{A}{2\sqrt{A^2+1}}} \qquad \text{and} \qquad A = \frac{2\Delta/\lambda - 1}{2\sqrt{2}}.$$

Larger trigonal distortions $|\Delta| \gg \lambda$ result in a more trivial state with no interesting orbital physics (the spin polarization of the hole is synonymous with that of the pseudospins, cf. red/blue colors in Fig. S5(a)), purely composed of $|\pm1, \mp\frac{1}{2}\rangle$ or $|0, \pm\frac{1}{2}\rangle$ states (depending on the sign of Δ). In order to determine the Hamiltonian parameters, we use wavefunctions to calculate several experimentally accessible properties. The longitudinal/transverse g-factors are given as

$$g_\parallel = 2|(1+k)c_1^2 - c_2^2| \qquad \text{and} \qquad g_\perp = 2c_2(c_2 - \sqrt{2}kc_1). \tag{6}$$

TABLE T4. Fit results of the single-ion + mean-field model against susceptibility, magnetization and ESR data.

Parameter	Value
λ	153.26 ± 0.03 meV
Δ	-77.94 ± 0.01 meV
k	0.9661 ± 0.0001
J_{MF}	1.71 ± 0.01 K
χ_a^0	$-9.2 \pm 4.8 \times 10^{-5}$ emu/mol
χ_c^0	$-6.5 \pm 1.2 \times 10^{-4}$ emu/mol

The single-ion magnetization can be calculated as

$$M_\alpha^{\mathrm{SI}}(\mathbf{H}, T) = \mu_{\mathrm{B}} \sum_i p_i \langle \psi_i | \, 2\hat{S}_\alpha - k\hat{\ell}_\alpha \, |\psi_i\rangle \,, \tag{7}$$

where p_i are Boltzmann weights and the uniform susceptibility

$$\chi_{\alpha\beta} = \frac{\partial M_\alpha}{\partial H_\beta} \tag{8}$$

is simply evaluated numerically. For a direct comparison to experiment, we include also a temperature independent susceptibility contribution $\chi^0_{\alpha\beta}$ as well as a mean-field Heisenberg exchange parameter J_{MF}. Each ion experiences a local field $\mathbf{H}_{\mathrm{MF}}$ proportional to the magnetization of its neighbors, determined self-consistently using the relation

$$\mathbf{M}^{\mathrm{MF}} = \mathbf{M}^{\mathrm{SI}}(\mathbf{H} + \mathbf{H}_{\mathrm{MF}}, T) \qquad \text{where} \qquad \mathbf{H}_{\mathrm{MF}} = -\frac{zJ_{\mathrm{MF}}}{(\mathbf{g}\mu_{\mathrm{B}})^2}\mathbf{M}^{\mathrm{MF}}. \tag{9}$$

Here $z = 6$ is the number of nearest neighbors, while $\mathbf{g} = \mathrm{diag}(g_\perp, g_\perp, g_\parallel)$ is the g-tensor. By simultaneously fitting the magnetization, susceptibility and ESR data for all field directions we obtain the parameter estimates summarized in Table T4. We note that in experiment we are highly sensitive to the ratio Δ/λ, while the overall energy scale only appears in the high-T curvature of inverse susceptibilities and becomes muddled together with χ^0_α. Therefore, one can still obtain reasonable agreement by changing both spin-orbit coupling and trigonal crystal field simultaneously within $\sim 20\%$, but $\Delta/\lambda \simeq -0.51$ remains tightly constrained.

III. ELECTRON SPIN RESONANCE

In Fig. S6 we present additional ESR data, characterizing the temperature dependence of the spectra discussed in the main text. For both $\mathbf{H} \parallel \mathbf{a}$ and $\mathbf{H} \parallel \mathbf{c}$ configurations, the resonance line is visible as a dip in optical transmission, which becomes broader and more intense upon cooling. In both cases the Larmor frequency, i.e. the g-factor, does not seem to change with temperature.

IV. MAGNETOELASTICS & EXCHANGE STRICTION

Let us recall the exchange striction mechanism commonly responsible for magnetoelastic effects near phase transitions in magnetic insulators [9]. Exchange integrals critically depend on the distances between magnetic ions, as well as their angles with the ligands mediating the bonding. For small changes, this can be linearized as

$$J(|\mathbf{r}_i - \mathbf{r}_j|) = J(|\mathbf{r}^0_{ij}|) + \rho_{ij} \frac{\partial J}{\partial \rho_{ij}}\bigg|_{\rho_{ij}=0} + \mathcal{O}(\rho_{ij}^2) \tag{10}$$

where $\rho_{ij} = |\delta\mathbf{r}_{ij}|/|\mathbf{r}^0_{ij}|$ is a normalized bond displacement induced by some lattice strain ε_Γ. This allows us to write down a simple "bond-phonon" [10, 11] magnetoelastic Hamiltonian

$$\mathcal{H}_{\mathrm{m-e}} = \sum_{\langle i,j\rangle} \left[(J_0 - g\rho_{ij})\mathbf{S}_i \cdot \mathbf{S}_j + \frac{k}{2}\rho_{ij}^2 \right] \tag{11}$$

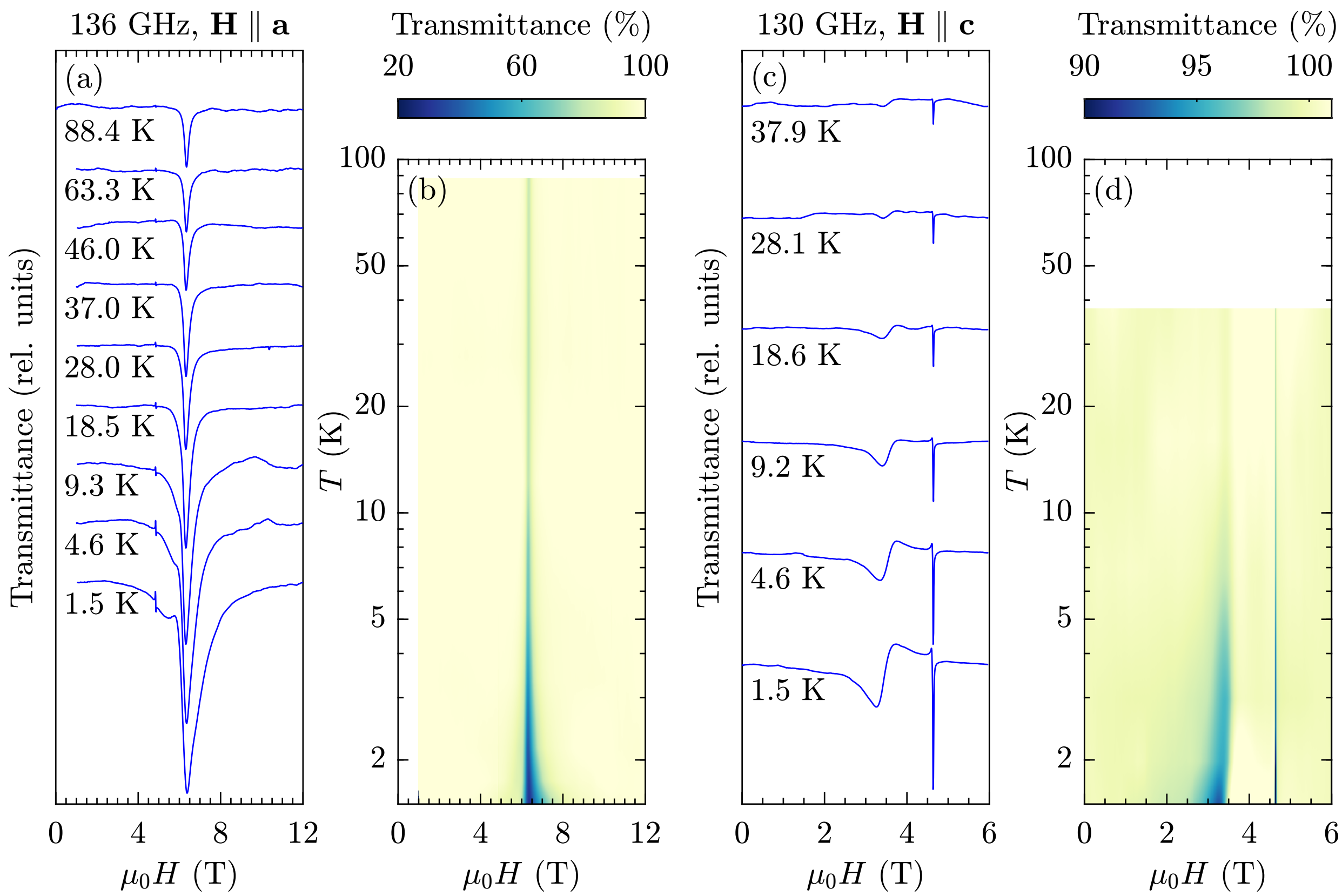

FIG. S6. Temperature dependence of electron spin resonance excitations. (a,c) Fixed frequency ESR spectra at various temperatures in $\mathbf{H} \parallel \mathbf{a}$ and $\mathbf{H} \parallel \mathbf{c}$ experimental configurations. (b,d) False colorplots of the optical transmittance against temperature and magnetic field.

where k is an elastic modulus and the coupling constant $g \equiv -\frac{\partial J}{\partial \rho_{ij}}$ sets the strength of spin-lattice effects. In equilibrium we have $\partial \mathcal{H}/\partial \rho_{ij} = 0$, which yields $\langle\langle \rho_{ij} \rangle\rangle = \frac{g}{k} \langle \mathbf{S}_i \cdot \mathbf{S}_j \rangle$ for the normalized changes in bond length. As spin correlations develop below T_{N}, each bond acquires an additional stiffness contribution $\propto \langle \mathbf{S}_i \cdot \mathbf{S}_j \rangle$ and the lattice contracts to accommodate this. In principle one can "integrate out" the phonon terms in the Hamiltonian, which produces an effective bi-quadratic term $\sim b(\mathbf{S}_i \cdot \mathbf{S}_j)^2$ in the spin model where $b_{\mathrm{eff}} = -g^2/k$.

**Mode**	δr_{12}	δr_{13}	δr_{23}
A_1	-1	-1	-1
E(1)	1	$-\frac{1}{2}$	$-\frac{1}{2}$
E(2)	0	$\frac{\sqrt{3}}{2}$	$-\frac{\sqrt{3}}{2}$

TABLE T5. Bond elongation δr_{ij} for D_3 symmetry strains (i.e. vibrational modes) A_1 and E of an equilateral triangle.

A. Estimating the Coupling Constants

Based on the lattice strains $\Delta l/l$ determined in zero field, we attempt to estimate the magnetoelastic coupling constants. To check which type of distortions may be realized, we look at the symmetry strains, irreps of the fundamental vibrational modes that may deform the crystal [12]. Restricting ourselves to the case of $\mathbf{k} = 0$ distortions, the problem simplifies to that of the D_3 point group for an equilateral triangle. There are two relevant irreps: The trivial A_1 "breathing" mode, expanding/contracting the entire triangle, and a two-fold degenerate E mode allowing e.g. for a $J - J'$ isosceles distortion.

The simple A_1 mode should be always active, since the magnetic energy depends linearly on the strain $\varepsilon_{\mathrm{A}_1}$, e.g.

$$E_{\mathrm{mag}}(\varepsilon) \approx -\frac{3}{2}(J - g_{\mathrm{A}_1} \langle\langle \varepsilon_{\mathrm{A}_1} \rangle\rangle) S^2 + \mathcal{O}(\varepsilon^2) \tag{12}$$

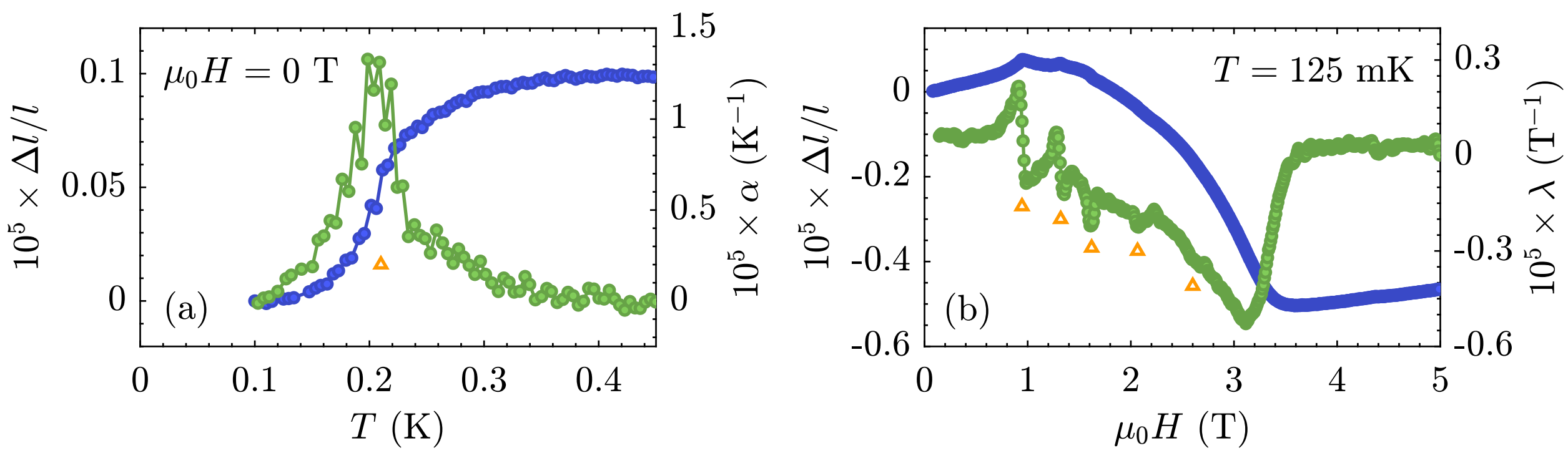

FIG. S7. Magnetoelastic effects in $(CD_3ND_3)_2NaRuCl_6$ perpendicular to the triangular planes. (a) Temperature dependent sample dilation $\Delta l/l$ along the c-axis, together with its numerical derivative, the thermal expansion coefficient $\alpha = \frac{1}{l}\frac{\partial \Delta l}{\partial T}$. (b) Changes in sample length in a magnetic field $\mathbf{H} \parallel \mathbf{c}$, along with the magnetostriction coefficient $\lambda = \frac{1}{\mu_0 l}\frac{\partial \Delta l}{\partial H}$. Orange triangles in both panels denote the anomalies in $\alpha(T)$ and $\lambda(H)$, in agreement with the phase transitions discussed in the main text.

for a classical Heisenberg model on the triangular lattice. As for the "real" low-temperature structure, we take $\langle \mathbf{S}_i \cdot \mathbf{S}_j \rangle \approx -\frac{1}{2} \langle\langle S \rangle\rangle^2$ with a reduced ordered moment $\langle\langle S \rangle\rangle \approx 0.21$ [13–15] and ignore the small incommensurability (the angles between adjacent spins remain close to 120°). The relevant stiffness constant is $k = \frac{V_0(c_{11}+c_{12})}{2}$, where V_0 is the unit cell volume, the longitudinal sound velocity $v_{11} = \sqrt{c_{11}/\rho} \simeq 2.1$ km/s is determined from ultrasound and ρ represents the mass density. Here we make the rough assumption $k \sim \frac{3V_0 c_{11}}{4}$ based on the elastic stability conditions $0 < c_{12} < c_{11}$ for hexagonal crystals [16]. Given the strain $\langle\langle \varepsilon_{A_1} \rangle\rangle \equiv \Delta l/l \sim -5 \times 10^{-6}$ observed in our zero field thermal expansion data, we estimate the spin-lattice coupling constant

$$g_{A_1} = \frac{k \Delta l/l}{\langle\langle \mathbf{S}_i \cdot \mathbf{S}_j \rangle\rangle} \sim 40 \text{ K}. \tag{13}$$

B. $J - J'$ Magnetoelastic Distortion

While our x-ray data confirm the trigonal crystal structure down to T_N, they cannot rule out the possibility of a "dynamic" $J - J'$ isosceles distortion coupled to the magnetic order parameter. Such a monoclinic lattice distortion would correspond to the E(1) vibrational mode and may coexist with A_1 below T_{N2} on a similar scale $\lesssim 10^{-6}$. Starting from a simple Heisenberg picture, the triangular lattice would be deformed into inequivalent "chain" and "zig-zag" couplings with renormalized exchanges $J_0 \to J = J_0(1-\epsilon)$ and $J_0 \to J' = J_0(1+\frac{\epsilon}{2})$ respectively (here we define $\epsilon \equiv g_{E_1} \langle\langle \varepsilon_{E_1} \rangle\rangle / J_0$ in the above exchange-striction framework). This affects both the classical propagation vector and the ground state energy, resulting in

$$|\mathbf{q}(\epsilon)| = 2\cos^{-1}\left(-\frac{J'}{2J}\right) = 2\cos^{-1}\left(-\frac{1+\epsilon/2}{2(1-\epsilon)}\right) = \frac{4\pi}{3} + \sqrt{3}\epsilon + \mathcal{O}(\epsilon^2) \tag{14}$$

$$E_{\text{mag}}(\epsilon) = J\cos(q) + 2J'\cos\left(\frac{q}{2}\right) = -\frac{3}{2}J_0 S^2 - \frac{9}{8}J_0 S^2 \epsilon^2 + \mathcal{O}(\epsilon^3). \tag{15}$$

Qualitatively, this approach can explain the IC peak positions observed in zero field as due to three structural domains with an underlying spiral ground state. However, there are several problems with such a theory. First off, a lattice distortion $\langle\langle \varepsilon_{E(1)} \rangle\rangle \sim 10^{-6}$ consistent with experiment would require a *huge* coupling constant $g_{E(1)} \sim 10^3 g_{A_1}$ to explain the IC shift $\delta q/q = 3\sqrt{3} g_{E(1)} \langle\langle \varepsilon_{E(1)} \rangle\rangle / 4\pi J_0 \sim 2\%$ of magnetic Bragg peaks observed experimentally. This in turn would necessitate a stiffness constant $k_{E(1)} = \frac{v(c_{11}-c_{12})}{2}$ three orders of magnitude smaller, i.e. $c_{11} - c_{12} \sim 10^{-3} c_{11}$. What's even more problematic is that the classical magnetic energy gain from the distortion is *quadratic* in the strain at lowest order, not linear. The latter is directly competing against the cost in elastic energy (also quadratic), which means an E-type distortion is generally not expected to appear.

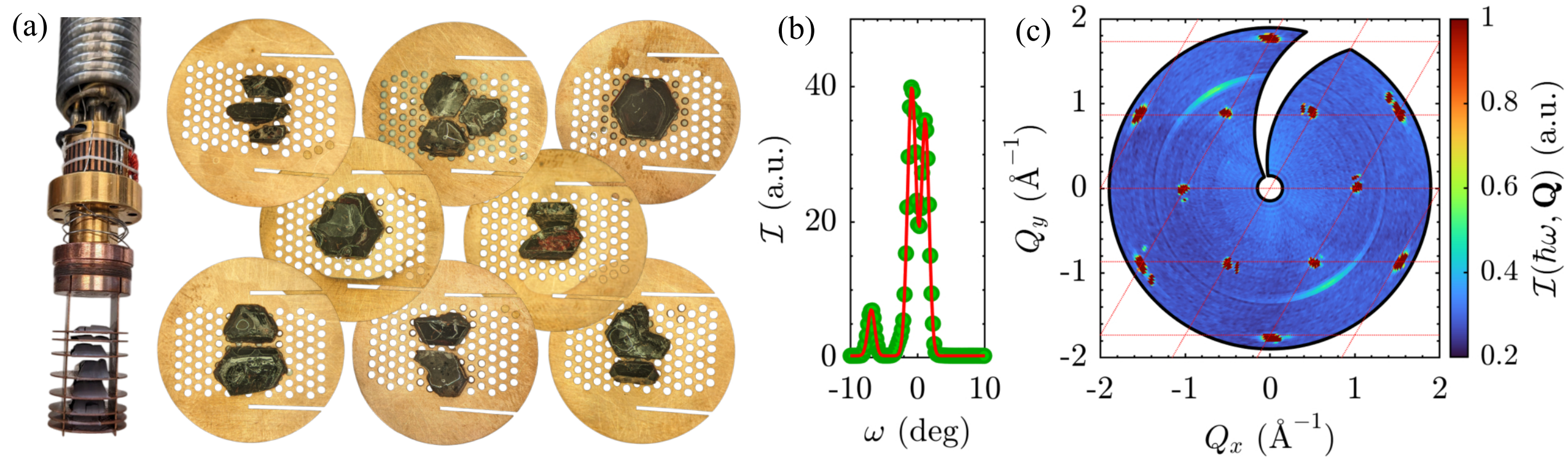

FIG. S8. (a) The probe used for neutron spectroscopy, containing 16 single crystals coaligned in the ab scattering plane. (b) A cut through the $\mathbf{Q} = (1, 0, 0)$ nuclear bragg peak. (c) Elastic neutron scattering intensity in the $(hk0)$ plane taken at $T \simeq 1$ K. The powder line visible at $|\mathbf{Q}| \sim 1.3$ Å^{-1} originates from teflon.

C. Dilatometry in $\mathbf{E} \parallel \mathbf{c}$ Geometry

In Fig. S7 we present additional dilatometry data in the $\mathbf{H} \parallel \mathbf{E} \parallel \mathbf{c}$ configuration, showing the length changes *perpendicular* to the triangular planes. We observe the same phase transitions discussed in the main text, both against temoerature (thermal expansion) and magnetic field (magnetostriction). Note that the overall scale of these magnetoelastic effects is roughly an order of magnitude weaker than within the triangular plane, as expected for a quasi-2D system.

V. INELASTIC NEUTRON SCATTERING

In the following, we provide further details about the sample and fitting procedure employed for the neutron spectroscopy experiment carried out at THALES (ILL). Fig. S8(a) shows a picture of the coaligned probe, composed of 16 crystals fixed to a copper holder with cytop glue and teflon. The total sample mass amounts to $m = 1.9$ g. The quality of the alignment can be seen in Fig. S8(b,c), with a sample mosaic of roughly 3° FWHM. One can also spot a small crystallite, slightly misaligned by 6.9° in the $(hk0)$ plane and contributing about 7(2)% to the total scattering intensity.

To reconstruct the spin-wave dispersion relation presented in the main text, we masked out spurious intensity from inelastically re-scattered Bragg peaks ("Currat-Axe spurions"), subtracted a constant background and normalized the data by the magnetic form factor. We then averaged over the various equivalent reciprocal space paths presented in Fig. S9(a-c) and performed Gaussian fits to constant-$\mathbf{Q}$ cuts in order to extract the dispersion points $\hbar\omega_{\mathbf{Q}}$.

These observations are compared to the magnon dispersion of a fully polarized ferromagnetic state in a least squared fitting procedure. There is only one branch (propagation vector $\mathbf{q} = \Gamma$) and spin-wave theory is exact [17], making this the perfect regime to extract exchange parameters. For longitudinal fields $\mathbf{H} \parallel \mathbf{c}$, the magnon dispersion for the general anisotropic spin Hamiltonian of Eq. 1 is given as

$$\begin{aligned}\hbar\omega(\mathbf{Q}) &= 2S\sqrt{A_{\mathbf{Q}}^2 - |B_{\mathbf{Q}}|^2} \\ A_{\mathbf{Q}} &= g_c\mu_B B_z - 3J\Delta + J\gamma_1(\mathbf{Q}) \\ B_{\mathbf{Q}} &= J_{\pm\pm}\left(\gamma_2(\mathbf{Q}) + \sqrt{3}i[\cos(2\pi k) - \cos(2\pi(h+k))]\right),\end{aligned} \tag{16}$$

where

$$\begin{aligned}\gamma_1(\mathbf{Q}) &= \cos(2\pi h) + \cos(2\pi k) + \cos(2\pi(h+k)) \\ \gamma_2(\mathbf{Q}) &= -2\cos(2\pi h) + \cos(2\pi k) + \cos(2\pi(h+k)).\end{aligned} \tag{17}$$

In this geometry, $J_{z\pm}$ is absent entirely from the spin-wave dispersion. Furthermore, at high fields we have $|A_{\mathbf{Q}}| \gg |B_{\mathbf{Q}}|$, i.e. $\hbar\omega(\mathbf{Q}) \approx 2S|A_{\mathbf{Q}}|$ and we are mainly sensitive to the XXZ-type couplings with only a weak dependence on $|J_{\pm\pm}|$. In the Heisenberg limit ($\Delta = 1, J_{\pm\pm} = J_{z\pm} = 0$), Eq. 16 simplifies to

$$\hbar\omega(\mathbf{Q}) = 2S\left(g_c\mu_B B_z - 3J + J\gamma_1(\mathbf{Q})\right). \tag{18}$$

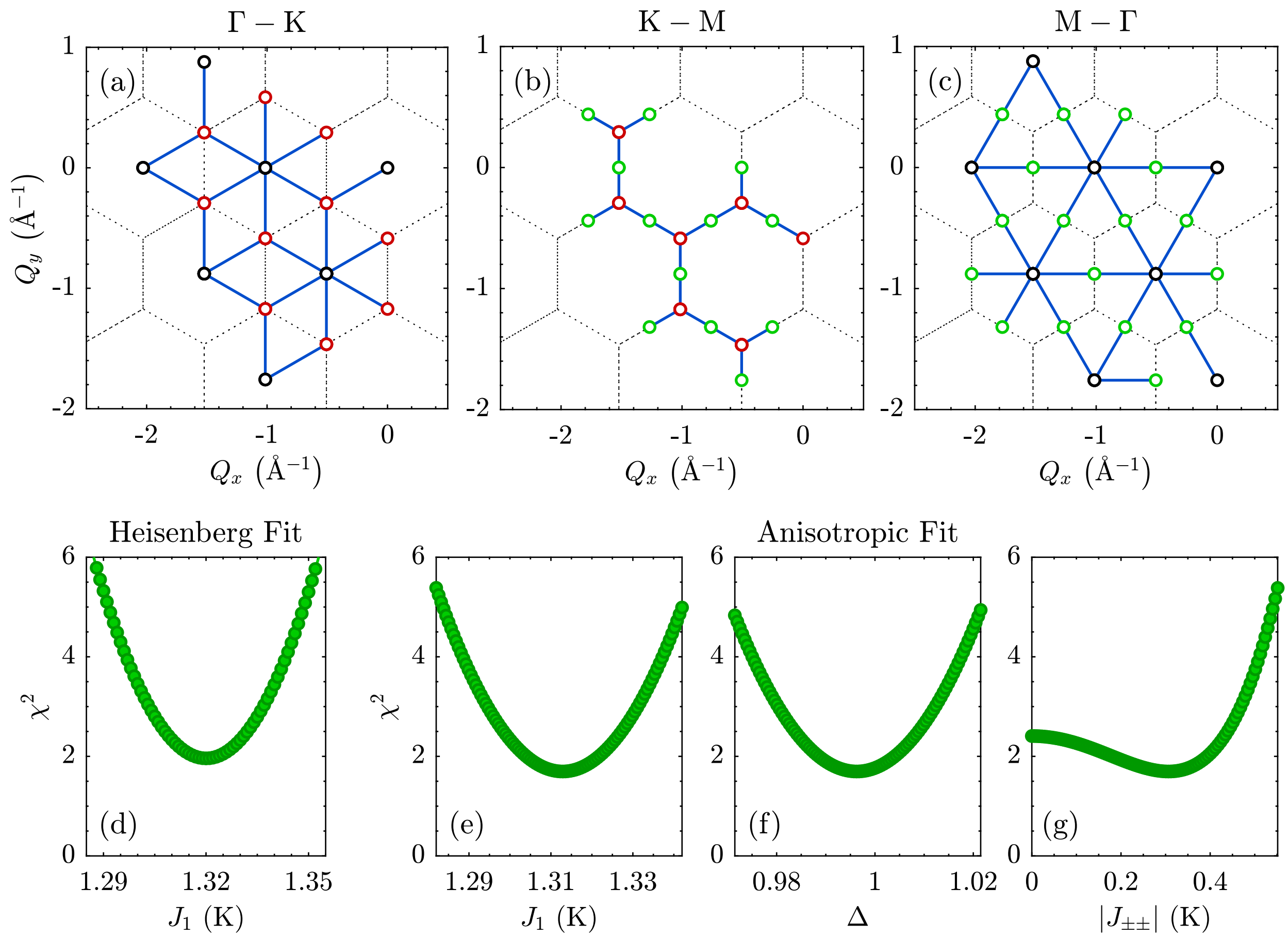

FIG. S9. (a-c) Schematic showing the equivalent reciprocal space paths along Γ - K, K - M and M - Γ, used to plot the averaged excitation spectrum in the main text. Brillouin zone boundaries are indicated as black dotted lines. (d) χ^2 goodness-of-fit metric of the extracted dispersion relation to a nearest-neighbor Heisenberg model on the 2D triangular lattice. (e-g) Analogous χ^2 plots for the general anisotropic spin model of Eq. 1.

The χ^2 goodness-of-fit metrics, obtained by fitting the experimental dispersion relation to the Heisenberg and the anisotropic model, are depicted in Fig. S9(d-g).

VI. COMMENSURATE MAGNETIC STRUCTURES

Here we discuss the nature of the commensurate magnetic structures in $(CD_3ND_3)_2NaRuCl_6$. Their essential physics can be understood through a Heisenberg model on the triangular lattice, with the addition of a small AFM inter-plane coupling $J_c > 0$ [18]. Given the observed propagation vector $\mathbf{q} = (1/3, 1/3, 1/2)$, we find the ground state by considering all possible six-sublattice spin configurations and resorting to semi-classical energy minimization. At $T = 0$, the classical degeneracy is lifted only by quantum fluctuations (i.e. order-by-disorder selection), favoring the "most collinear" states with the softest excitation spectrum [19, 20]. On a phenomenological level, one can emulate this by adding a small bi-quadratic coupling $\sim b(\mathbf{S}_i \cdot \mathbf{S}_j)^2$ to the Hamiltonian [21–23]. In fact, such a term can be derived rigorously through real space perturbation theory (RSPT), by calculating the leading order corrections to the "mean field" (i.e. classical) ground state energy. For the triangular lattice Heisenberg antiferromagnet, the second order RSPT correction is given as [24]

$$\delta E_2 = \sum_n \frac{\langle 0|\, \hat{V}\, |n\rangle \langle n|\, \hat{V}\, |0\rangle}{E_0 - E_n} = -\frac{J^2 S^2}{8 H_{\mathrm{loc}}} \sum_{\langle i,j \rangle} (1 - \cos\theta_{ij})^2 \tag{19}$$

where $H_{\mathrm{loc}} = 3JS$ is the local field acting on each spin, resulting in $b_{\mathrm{eff}} \equiv -J/(24S^3)$. Since this energy correction amounts to an effective Hamiltonian operating on the ground state manifold of classical vector spins $\mathbf{n}_i = \mathbf{S}_i/S$, the bi-quadratic term $\sim \cos^2\theta_{ij} = (\mathbf{n}_i \cdot \mathbf{n}_j)^2$ can show up even for $S = 1/2$ quantum systems.

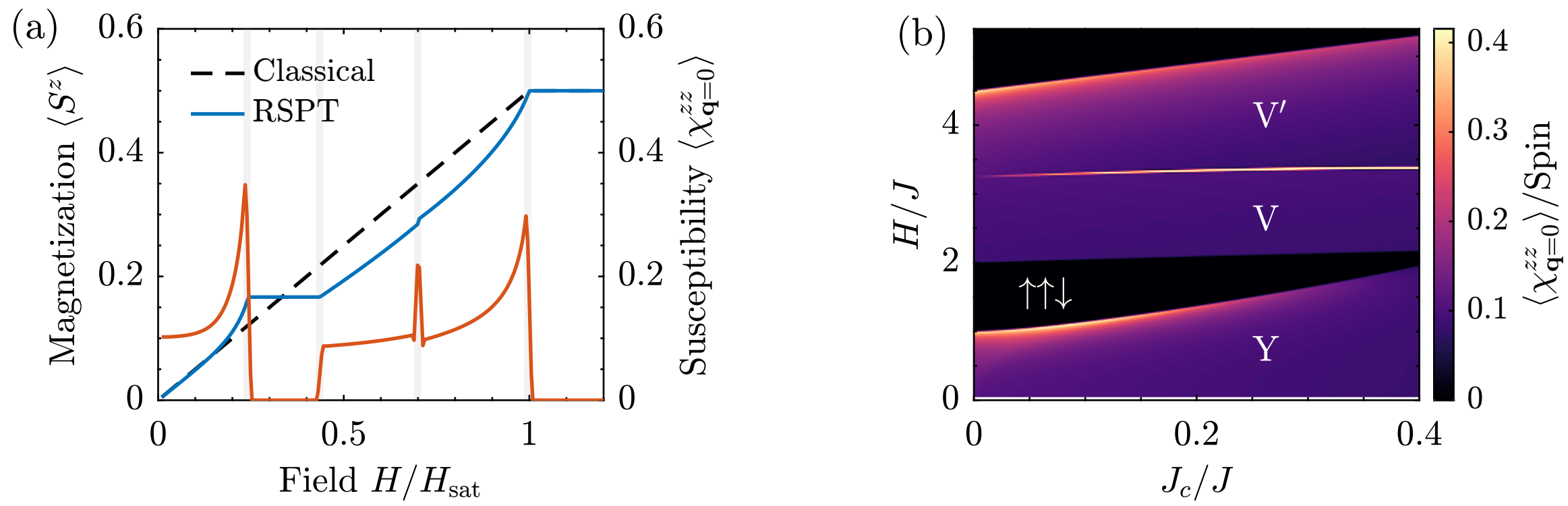

FIG. S10. Effects of inter-plane coupling on the magnetic structures for the Heisenberg triangular lattice antiferromagnet. (a) Magnetization curve and its numerical derivative for $J_c = 0.1J$, obtained from semi-classical model discussed in the text. (b) $T = 0$ ground state phase diagram in the same approach, visualized as false color plot of the uniform magnetic susceptibility $\chi(J_c, H)$. Both panels clearly show the presence of an additional phase transition between the V and V′ states.

We find the optimal six-sublattice ground state by minimizing the following semi-classical Hamiltonian

$$\mathcal{H} = \sum_{\langle i,j \rangle \in \Delta} \left[J \mathbf{S}_i \cdot \mathbf{S}_j + b_{\rm eff}(S^2 - \mathbf{S}_i \cdot \mathbf{S}_j)^2 \right] + J_c \sum_{\langle i,j \rangle \| c} \mathbf{S}_i \cdot \mathbf{S}_j - \sum_i \mathbf{S}_i \cdot \mathbf{H} \tag{20}$$

as a function of the inter-plane coupling J_c and magnetic field H, resulting in the spin configurations discussed in the main text. The phase diagram, as well as magnetization curves for a reasonable parameter choice $J_c = 0.1J$, are shown in Fig. S10. There are two differences compared to the pure 2D model: Firstly, the inter-plane term becomes frustrated by the field, resulting in a mixed FM-AFM stacking with both $\mathbf{q} = (1/3, 1/3, 1/2)$ and $2\mathbf{q} = (1/3, 1/3, 0)$ Fourier components. Furthermore, the stacking preferred just above the plateau differs from the one close to saturation, which gives rise to a first-order transition around $H_c \approx 0.7H_{\rm sat}$, above/below which the choice of minority spin is uniform/staggered between layers. The evolution between phases Y - ↑↑↓ - V - V′ appears to be highly robust with respect to J_c, until the collinear magnetization plateau eventually becomes suppressed for large $J_c \gtrsim 0.5J$.

Finally, we remark on the effects of other perturbing terms on these magnetic structures. The presence of several IC phases in $(CD_3ND_3)_2NaRuCl_6$ clearly points to significant bond-dependent interactions or magnetoelastic effects. But towards the center of the phase diagram, the energy gain through order-by-disorder is maximized, surpassing those other contributions and leading to a lock-in to the semi-classical commensurate states discussed above [22, 25]. In absence of a lattice distortion, magnetoelastic effects can only contribute a tiny bi-quadratic term ($\sim$ 20 times smaller than the one obtained from RSPT, see Sec. IV) and should be irrelevant. The bond anisotropies would favor collinear four-sublattice stripe order [26]. Below some critical exchange ratio, their main effect would be to renormalize the critical fields H_c and break the $U(1)$ rotational symmetry, pinning the staggered in-plane ordered moment to some discrete orientation.

VII. $\mathbb{Z}_2$ VORTEX CRYSTAL PHASE

For sake of completeness, we reproduce here the expected 2D spin configuration for the $\mathbb{Z}_2$ vortex crystal phase [27–29]. Taking the cubic axes, one can equivalently re-write the spin Hamiltonian of Eq. 1 as a $J_0 - K - \Gamma - \Gamma'$ model [26]. The vortex crystal has been predicted to exist in the Heisenberg-Kitaev limit (i.e. $\Gamma = \Gamma' = 0$), where

$$\mathcal{H} = J_0 \sum_{\langle i,j \rangle} \mathbf{S}_i \cdot \mathbf{S}_j + K \sum_{\gamma \| \langle i,j \rangle} S_i^\gamma S_j^\gamma \tag{21}$$

and the coordinate transformation amounts to $J_0 = J + 6J_{\pm\pm}, K = -6J_{\pm\pm}$ with the constraints $\Delta = 1$ (confirmed experimentally in $(CD_3ND_3)_2NaRuCl_6$) and $J_{z\pm} = 2\sqrt{2}J_{\pm\pm}$ (yet to be determined). In momentum space, the classical HK-Hamiltonian has three eigenvalues at

$$\mathcal{J}^{\gamma\gamma}(\mathbf{q}) = K \cos(\mathbf{q} \cdot \hat{\mathbf{e}}_\gamma) + J \sum_{\alpha = x,y,z} \cos(\mathbf{q} \cdot \hat{\mathbf{e}}_\alpha), \tag{22}$$

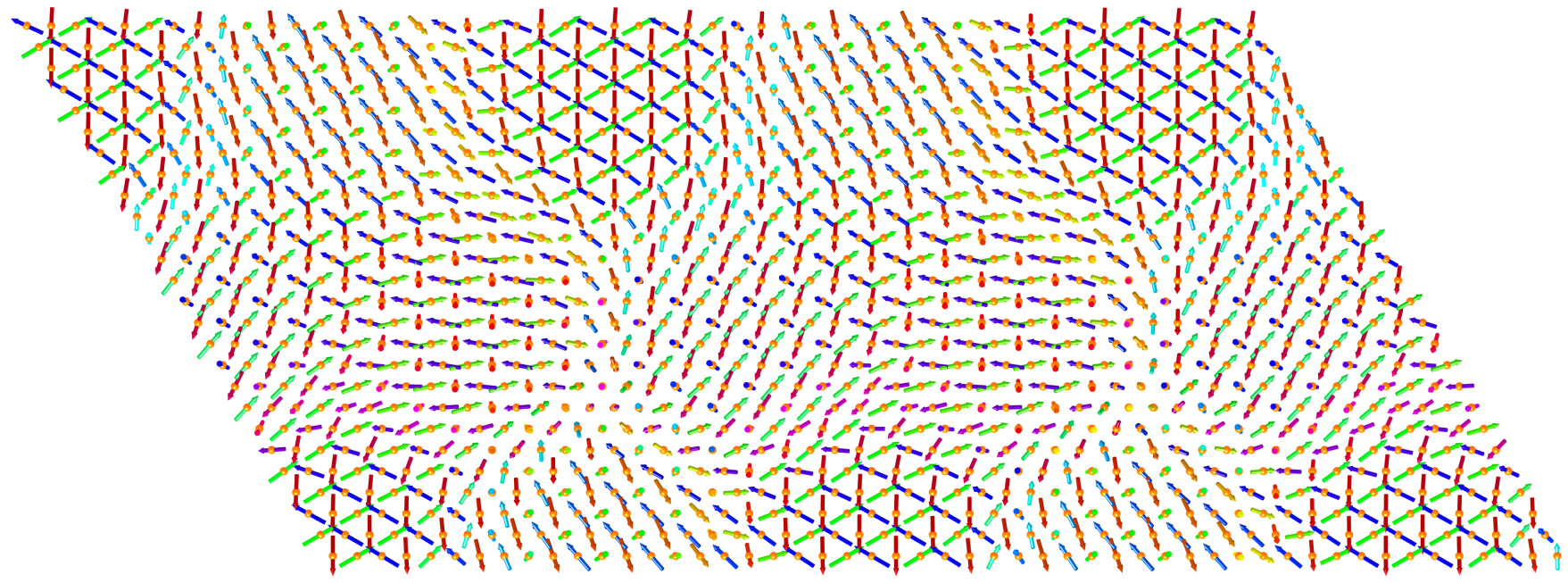

FIG. S11. Sketch of the 2D spin configuration for a $\mathbb{Z}_2$ vortex crystal in the Heisenberg-Kitaev model, with parameters $K/J_0 = -0.3$. The *hsv*-color of each spin indicates its rotation angle in the triangular plane.

i.e. each spin component $\gamma \in (x, y, z)$ prefers a different ordering vector. We obtain three sets of incommensurate positions $\pm\mathbf{q}^\gamma$, along the Γ-K direction in momentum space. These minima move towards the Γ (M)-point for positive (negative) K, where

$$|\mathbf{q}| = 1 - \frac{1}{\pi}\cos^{-1}\left(\frac{J}{2(J+K)}\right) \quad \Longrightarrow \quad |\delta q| \approx \frac{1}{\sqrt{3}\pi}\frac{|K|}{|J+\frac{7}{6}K|}. \tag{23}$$

A simple superposition of three such incommensurate spirals will not be a good ground state - the moment size would be strongly modulated. Instead the system would turn anharmonic, introducing additional Fourier components to retain a semi-classical equal moment structure. The resulting ground state preserves threefold symmetry and locally mimics the "parent" 120° structure, but exhibits a superlattice of $\mathbb{Z}_2$ vortices on larger length scales. Up to second harmonics, the magnetic structure may be approximated as

$$S^\gamma(\mathbf{r}) = \frac{4S}{3\sqrt{3}}\mathrm{Re}\left[e^{i[\mathbf{q}_\gamma\cdot\mathbf{r}+\phi]} + \frac{1}{4}\sum_{\alpha\neq\gamma} e^{i[(\mathbf{q}_\gamma+\mathbf{q}_\alpha)\cdot\mathbf{r}+\phi]}\right] \tag{24}$$

with a phase shift $\phi = 2\pi n/3$ between sublattices, visualized in Fig. S11.

[1] P. Vishnoi, J. L. Zuo, T. A. Strom, G. Wu, S. D. Wilson, R. Seshadri, and A. K. Cheetham, Structural Diversity and Magnetic Properties of Hybrid Ruthenium Halide Perovskites and Related Compounds, Angewandte Chemie International Edition **59**, 8974 (2020).

[2] A. Waśkowska, L. Gerward, J. Staun Olsen, W. Morgenroth, M. Mączka, and K. Hermanowicz, Temperature- and pressure-dependent lattice behaviour of $RbFe(MoO_4)_2$, Journal of Physics: Condensed Matter **22**, 055406 (2010).

[3] Y. Kajita, T. Nagai, S. Yamagishi, K. Kimura, M. Hagihala, and T. Kimura, Ferroaxial Transitions in Glaserite-Type $Na_2BaM(PO_4)_2$ (M = Mg, Mn, Co, and Ni), Chemistry of Materials **36**, 7451 (2024).

[4] L. Woodland, R. Okuma, J. R. Stewart, C. Balz, and R. Coldea, From continuum excitations to sharp magnons via transverse magnetic field in the spin-$\frac{1}{2}$ ising-like triangular lattice antiferromagnet $Na_2BaCo(PO_4)_2$, Phys. Rev. B **112**, 104413 (2025).

[5] A. J. Hearmon, F. Fabrizi, L. C. Chapon, R. D. Johnson, D. Prabhakaran, S. V. Streltsov, P. J. Brown, and P. G. Radaelli, Electric Field Control of the Magnetic Chiralities in Ferroaxial Multiferroic $RbFe(MoO_4)_2$, Physical Review Letters **108**, 237201 (2012).

[6] A. Zelenskiy, J. A. Quilliam, A. Ya. Shapiro, and G. Quirion, Magnetic phases of the S=5/2 triangular-lattice antiferromagnet $RbFe(MO_4)_2$ as determined by ultrasound velocity measurements, Physical Review B **103**, 224422 (2021).

[7] A. Abragam and B. Bleaney, *Electron Paramagnetic Resonance of Transition Ions*, 1st ed., Oxford classic texts in the physical sciences (Oxford University Press, 1970).

[8] Y. Kubota, H. Tanaka, T. Ono, Y. Narumi, and K. Kindo, Successive magnetic phase transitions in $RuCl_3$: XY-like frustrated magnet on the honeycomb lattice, Physical Review B **91**, 094422 (2015).

[9] M. E. Lines, Elastic properties of magnetic materials, Physics Reports **55**, 133 (1979).

[10] K. Penc, N. Shannon, and H. Shiba, Half-Magnetization Plateau Stabilized by Structural Distortion in the Antiferromagnetic Heisenberg Model on a Pyrochlore Lattice, Physical Review Letters **93**, 197203 (2004).

[11] D. L. Bergman, R. Shindou, G. A. Fiete, and L. Balents, Models of degeneracy breaking in pyrochlore antiferromagnets, Physical Review B **74**, 134409 (2006).

[12] O. Tchernyshyov, R. Moessner, and S. L. Sondhi, Order by Distortion and String Modes in Pyrochlore Antiferromagnets, Physical Review Letters **88**, 067203 (2002).

[13] L. Capriotti, A. E. Trumper, and S. Sorella, Néel Order in the Triangular Heisenberg Model, Physical Review Letters **82**, 3899 (1999).

[14] W. Zheng, J. O. Fjærestad, R. R. P. Singh, R. H. McKenzie, and R. Coldea, Excitation spectra of the spin-$\frac{1}{2}$ triangular-lattice Heisenberg antiferromagnet, Physical Review B **74**, 224420 (2006).

[15] S. R. White and A. L. Chernyshev, Néel Order in Square and Triangular Lattice Heisenberg Models, Physical Review Letters **99**, 127004 (2007).

[16] F. Mouhat and F.-X. Coudert, Necessary and sufficient elastic stability conditions in various crystal systems, Physical Review B **90**, 224104 (2014).

[17] R. Coldea, D. A. Tennant, K. Habicht, P. Smeibidl, C. Wolters, and Z. Tylczynski, Direct Measurement of the Spin Hamiltonian and Observation of Condensation of Magnons in the 2D Frustrated Quantum Magnet Cs_2CuCl_4, Physical Review Letters **88**, 137203 (2002).

[18] D. Yamamoto, G. Marmorini, and I. Danshita, Microscopic Model Calculations for the Magnetization Process of Layered Triangular-Lattice Quantum Antiferromagnets, Physical Review Letters **114**, 027201 (2015).

[19] J. Villain, R. Bidaux, J.-P. Carton, and R. Conte, Order as an effect of disorder, Journal de Physique **41**, 1263 (1980).

[20] A. V. Chubukov and D. I. Golosov, Quantum theory of an antiferromagnet on a triangular lattice in a magnetic field, Journal of Physics: Condensed Matter **3**, 69 (1991).

[21] M. T. Heinilä and A. S. Oja, Selection of the ground state in type-I fcc antiferromagnets in an external magnetic field, Physical Review B **48**, 7227 (1993).

[22] C. Griset, S. Head, J. Alicea, and O. A. Starykh, Deformed triangular lattice antiferromagnets in a magnetic field: Role of spatial anisotropy and Dzyaloshinskii-Moriya interactions, Physical Review B **84**, 245108 (2011).

[23] G. Koutroulakis, T. Zhou, Y. Kamiya, J. D. Thompson, H. D. Zhou, C. D. Batista, and S. E. Brown, Quantum phase diagram of the $S = \frac{1}{2}$ triangular-lattice antiferromagnet $Ba_3CoSb_2O_9$, Physical Review B **91**, 024410 (2015).

[24] M. E. Zhitomirsky, Real-space perturbation theory for frustrated magnets: Application to magnetization plateaus, Journal of Physics: Conference Series **592**, 012110 (2015).

[25] R. Chen, H. Ju, H.-C. Jiang, O. A. Starykh, and L. Balents, Ground states of spin-1/2 triangular antiferromagnets in a magnetic field, Physical Review B **87**, 165123 (2013).

[26] P. A. Maksimov, Z. Zhu, S. R. White, and A. L. Chernyshev, Anisotropic-Exchange Magnets on a Triangular Lattice: Spin Waves, Accidental Degeneracies, and Dual Spin Liquids, Physical Review X **9**, 021017 (2019).

[27] I. Rousochatzakis, U. K. Rössler, J. van den Brink, and M. Daghofer, Kitaev anisotropy induces mesoscopic $\mathbb{Z}_2$ vortex crystals in frustrated hexagonal antiferromagnets, Physical Review B **93**, 104417 (2016).

[28] M. Becker, M. Hermanns, B. Bauer, M. Garst, and S. Trebst, Spin-orbit physics of $j = \frac{1}{2}$ Mott insulators on the triangular lattice, Physical Review B **91**, 155135 (2015).

[29] M. Li, N. B. Perkins, and I. Rousochatzakis, Collective spin dynamics of $\mathbb{Z}_2$ vortex crystals in triangular Kitaev-Heisenberg antiferromagnets, Physical Review Research **1**, 013002 (2019).
